# *MYC* transcription activation mediated by OCT4 as a mechanism of resistance to 13-*cis*RA-mediated differentiation in neuroblastoma

**DOI:** 10.1038/s41419-020-2563-4

**Published:** 2020-05-14

**Authors:** Sung-Jen Wei, Thinh H. Nguyen, In-Hyoung Yang, Dustin G. Mook, Monish Ram Makena, Dattesh Verlekar, Ashly Hindle, Gloria M. Martinez, Shengping Yang, Hiroyuki Shimada, C. Patrick Reynolds, Min H. Kang

**Affiliations:** 1 Cancer Center, Texas Tech University Health Sciences Center, Lubbock, TX 79430 USA; 2 Department of Pediatrics, Texas Tech University Health Sciences Center, Lubbock, TX 79430 USA; 3 Department of Physiology, The Johns Hopkins University School of Medicine, Baltimore, MD 21205 USA; 4 Department of Pathology, Texas Tech University Health Sciences Center, Lubbock, TX 79430 USA; 5 Biostatistics Department, Pennington Biomedical Research Center, Baton Rouge, LA 70808 USA; 6 Department of Pathology, School of Medicine, Stanford University, Stanford, CA 94305 USA; 7 Department of Internal Medicine, School of Medicine, Texas Tech University Health Sciences Center, Lubbock, TX 79430 USA

**Keywords:** Oncogenes, Paediatric cancer

## Abstract

Despite the improvement in clinical outcome with 13-*cis*-retinoic acid (13-*cis*RA) + anti-GD2 antibody + cytokine immunotherapy given in first response ~40% of high-risk neuroblastoma patients die of recurrent disease. *MYCN* genomic amplification is a biomarker of aggressive tumors in the childhood cancer neuroblastoma. MYCN expression is downregulated by 13-*cis*RA, a differentiating agent that is a component of neuroblastoma therapy. Although *MYC* amplification is rare in neuroblastoma at diagnosis, we report transcriptional activation of *MYC* medicated by the transcription factor OCT4, functionally replacing MYCN in 13-*cis*RA-resistant progressive disease neuroblastoma in large panels of patient-derived cell lines and xenograft models. We identified novel OCT4-binding sites in the *MYC* promoter/enhancer region that regulated *MYC* expression via phosphorylation by MAPKAPK2 (MK2). OCT4 phosphorylation at the S111 residue by MK2 was upstream of *MYC* transcriptional activation. Expression of OCT4, MK2, and c-MYC was higher in progressive disease relative to pre-therapy neuroblastomas and was associated with inferior patient survival. OCT4 or MK2 knockdown decreased c-MYC expression and restored the sensitivity to 13-*cis*RA. In conclusion, we demonstrated that high c-MYC expression independent of genomic amplification is associated with disease progression in neuroblastoma. MK2-mediated OCT4 transcriptional activation is a novel mechanism for activating the *MYC* oncogene in progressive disease neuroblastoma that provides a therapeutic target.

## Introduction

Neuroblastoma is a cancer of the sympathetic nervous system mostly occurring in young children^1^. High-risk patients are >18 months old with stage 4 disease, stage 3 with unfavorable histopathology, or any tumor with *MYCN* gene amplification^2^. Treatment of high-risk neuroblastoma with non-myeloablative (conventional) chemotherapy alone achieves an initial response in most patients, but eventually 80–90% of patients develop progressive disease (PD) refractory to further therapy^3^. Neuroblastoma can spontaneously mature to a benign tumor known as ganglioneuroma and a variety of agents have been shown to induce growth arrest and morphological differentiation (neurite outgrowth) of human neuroblastoma cell lines^4^. All-*trans* retinoic acid (ATRA) and isotretinoin (13-*cis*-retinoic acid = 13-*cis*RA)-induced morphological differentiation^5^, decreased *MYCN* expression, and decreased cell proliferation in both *MYCN* gene-amplified and non-amplified human neuroblastoma cells in vitro^6,7^.

A randomized Phase III clinical trial showed that intensive myeloablative therapy supported by autologous hematopoietic stem cell transplantation (ASCT) improved outcome for high-risk neuroblastoma relative to conventional chemotherapy^8–10^, and that outcome was further improved using 13-*cis*RA to treat minimal residual disease^11^ after ASCT^12^. A subsequent trial further improved survival by adding dinutuximab (a chimeric anti-GD2 antibody) and cytokines to post-ASCT maintenance therapy^13^. However, many high-risk neuroblastoma patients still ultimately die from progressive disease (PD)^1^. In this study, we sought to define molecular mechanisms of 13-*cis*RA resistance in neuroblastoma. We observed that c-MYC (but not MYCN) expression was consistently increased in neuroblastoma cell lines and patient-derived xenografts (PDX) established from clinical samples of PD neuroblastoma. Here we report a novel pathway of *MYC* transcriptional activation that confers resistance to 13-*cis*RA, is frequently activated in PD neuroblastoma, and may serve as a therapeutic target.

## Results

### *MYC* is transcriptionally activated in 13-*cis*RA-resistant neuroblastoma cells

Tumor biopsies are infrequently obtained for research from children with neuroblastoma at time of progressive disease (PD)^14–16^. Bone marrow and blood samples are collected by the Children’s Oncology Group/ALSF Childhood Cancer Repository (www.CCcells.org) to establish and bank low-passage patient-derived cell lines and PDXs, including lines established from patients who developed PD after therapy with 13-*cis*RA. These include pre-therapy at diagnosis (Dx) and post therapy PD models established from pre- and post therapy tumor samples from the same patient. Clinical annotation and validation of origin of the cell lines and PDXs used in this study are shown in Supplementary Tables 1 and 2.

**Fig. 1:**
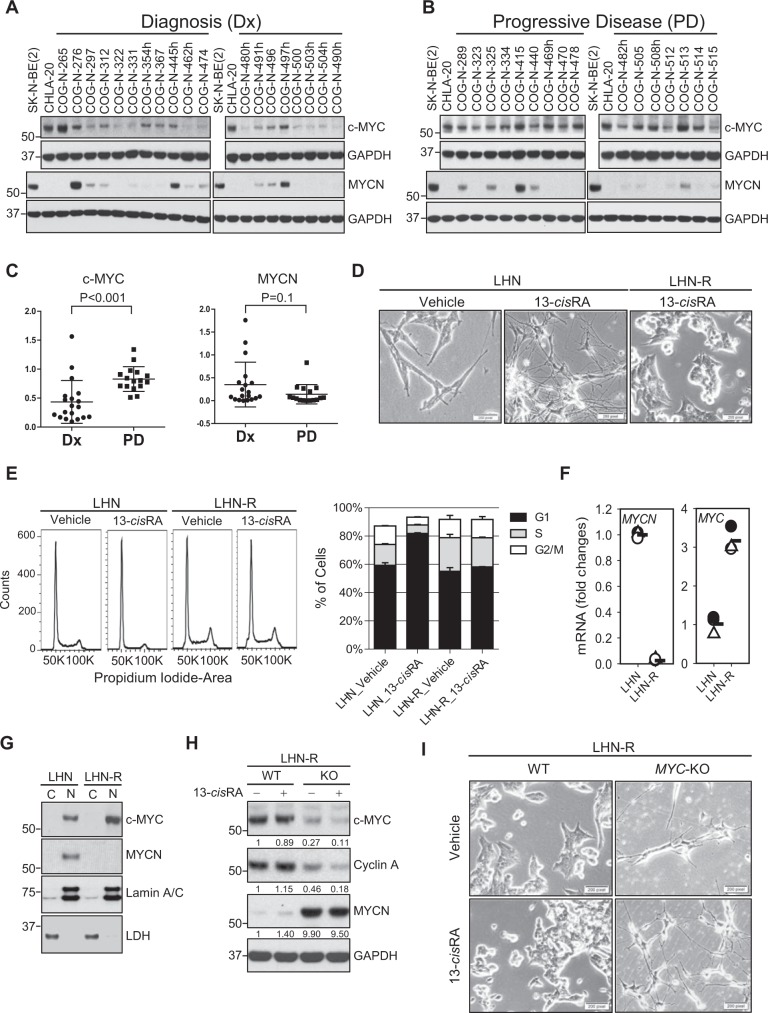
Expression of c-MYC was increased in patient-derived neuroblastoma cell lines of PD and conferred resistance to 13-*cis*RA. **a**, **b** Protein expression of c-MYC and MYCN in patient-derived neuroblastoma cell lines established from clinical samples collected at diagnosis (Dx) (**a**) and progressive disease (PD) (**b**). CHLA-20 and SK-N-BE(2) were used as the positive controls for c-MYC and MYCN. The results were replicated in independent experiments. **c** Dot plots quantitating immunoblots shown in **a** and **b**. The values obtained by densitometry were normalized in two ways by c-MYC in CHLA-20 and MYCN in SK-N-BE(2) per blot, and then by GAPDH for loading. **d** Morphology of SMS-LHN (LHN) cells that were treated with vehicle control or 5 μM 13-*cis*RA for 14 days and SMS-LHN-R (LHN-R, selected for resistance to 13-*cis*RA) cells. Cells were treated with 5 μM 13-*cis*RA (clinically achievable concentration) for 14 days. The observation was replicated in repeat experiments. Scale bar: 200 μM. **e** Cell cycle analyses of LHN and LHN-R cells treated with vehicle control or 5 μM 13-*cis*RA for 14 days (*p* < 0.01 for LHN, *p* = 0.07 for LHN-R). Triplicate samples of 10,000 events were analyzed. The error bars represent the SD. Left: representative histograms, right: percentage of cells in each phase of cell cycle. **f** Basal mRNA levels of *MYC* and *MYCN* in LHN and LHN-R cells. Relative quantitation (2^−ΔΔCT^) was used for the analyses of mRNA expression. In LHN-R relative to LHN, *MYCN* expression was significantly decreased while *MYC* expression was increased (*p* < 0.05) in three independent samples. Symbols: data, Bars: mean. **g** Expression of c-MYC and MYCN in subcellular fractions (C: cytosolic, N: nuclear) of LHN and LHN-R cells. LDH (cytosolic protein) and Lamin A/C (nuclear protein) were used as fractionation quality controls. The results were reproducible in a repeat experiment. **h** Effect of *MYC* knockout (KO) using CRISPR/Cas9 on Cyclin A, a downstream target of c-MYC in LHN-R cells. KO of *MYC* in both DNA strands was lethal to LHN-R cells, and thus the experiments were conducted in single KO cells. Morphological changes of *MYC* KO cells is shown in Supplementary Fig. S2b. The results were reproducible in a repeat experiment. **i** *MYC* knockout (KO) using a CRISPR/Cas9 system in LHN-R cells. *MYC* double knockout was lethal to LHN-R cells, and thus the experiments were conducted in *MYC* single knockout cells. The cells expressing wild-type *MYC* and *MYC* KO were treated with 13-*cis*RA at 5 mM for 14 days, and then the neurite outgrowth was observed under the bright-field microscope. Scale bar: 200 μM.

MYCN and c-MYC protein levels were compared in 19 Dx (Fig. 1a) and 16 PD (Fig. 1b) cell lines. c-MYC protein levels were significantly higher in PD models compared with Dx models (Fig. 1c). To determine whether increased c-MYC in PD cell lines might be due to the selective pressure of 13-*cis*RA treatment, the SMS-LHN (LHN) human neuroblastoma cell line (*MYCN* expression without genomic amplification)^17^ was treated with 13-*cis*RA, mimicking the clinical drug exposures obtained in children with neuroblastoma (5 μM for 2 wks of every 4 wks for 6 cycles). LHN responded initially to 13-*cis*RA with neurite outgrowth and decreased MYCN protein (Fig. 1d and Supplementary Fig. 1a). After six cycles of 13-*cis*RA, a resistant variant cell line (LHN-R) grew continuously during 13-*cis*RA treatment without showing neurite outgrowth (Fig. 1d) or cell cycle arrest (Fig. 1e). In LHN-R treated with 13-*cis*RA, mRNA and protein expression of c-MYC was increased while MYCN remained suppressed (Fig. 1f, g).

High c-MYC protein is seen in 11% of neuroblastoma at diagnosis (*MYC* genomic amplification seen in 1%) and has been associated with a poor clinical outcome^18^. Enhancer hijacking and focal enhancer amplification have been suggested as mechanisms for activating *MYC* expression in neuroblastoma^19^. However, the incidence of *MYC* transcriptional activation at PD and its molecular mechanisms remain unknown. As c-MYC was elevated in PD neuroblastoma cell lines and in those selected for resistance to *13-cis*RA, we sought to demonstrate that c-MYC overexpression confers resistance to 13-*cis*RA. Stable clones expressing wild-type c-MYC with 439 or 454 amino acids (AUG- and CUG-initiated) as well as deletion (∆72–209, encompassing *MYC* box 3) or point mutation (V409D, functionally critical in MAX dimerization) were created by transducing 4-hydroxytamoxifen (4-OHT)-inducible estrogen receptor (ER)-fusion constructs (Supplementary Fig. 1b) and confirmed exogenous protein levels for wild-type and mutant c-MYC (Supplementary Fig. 1c). Cyclin A, a c-MYC downstream target indicating c-MYC functionality, was detected in the nucleus of cells expressing c-MYC^439^, c-MYC^454^, and the V409D mutant after 13-*cis*RA treatment (Supplementary Fig. 1d). LHN cells expressing wild-type *MYC* did not respond to 13-*cis*RA treatment, whereas the deletion mutant showed significant cell cycle arrest by 13-*cis*RA (*p* < 0.01, Supplementary Fig. 1e).

To show the direct functional connection between c-MYC and Cyclin A expression, we used CRISPR/Cas9 to knockout *MYC* in LHN-R. *MYC* double knockout (KO) was lethal to LHN-R cells, and thus the experiments were conducted in *MYC* single KO cells. In the *MYC* KO cells, 13-*cis*RA decreased Cyclin A expression (Fig. 1h) and induced neurite outgrowth (Fig. 1i). *MYC* KO increased MYCN expression (Fig. 1h), and MYC overexpression resulted in the decrease in MYCN (Supplementary Fig. 1f). We noted that these data show that c-MYC overexpression causes resistance to 13-*cis*RA in neuroblastoma cell lines.

### OCT4 induced transcriptional activation of *MYC*

**Fig. 2:**
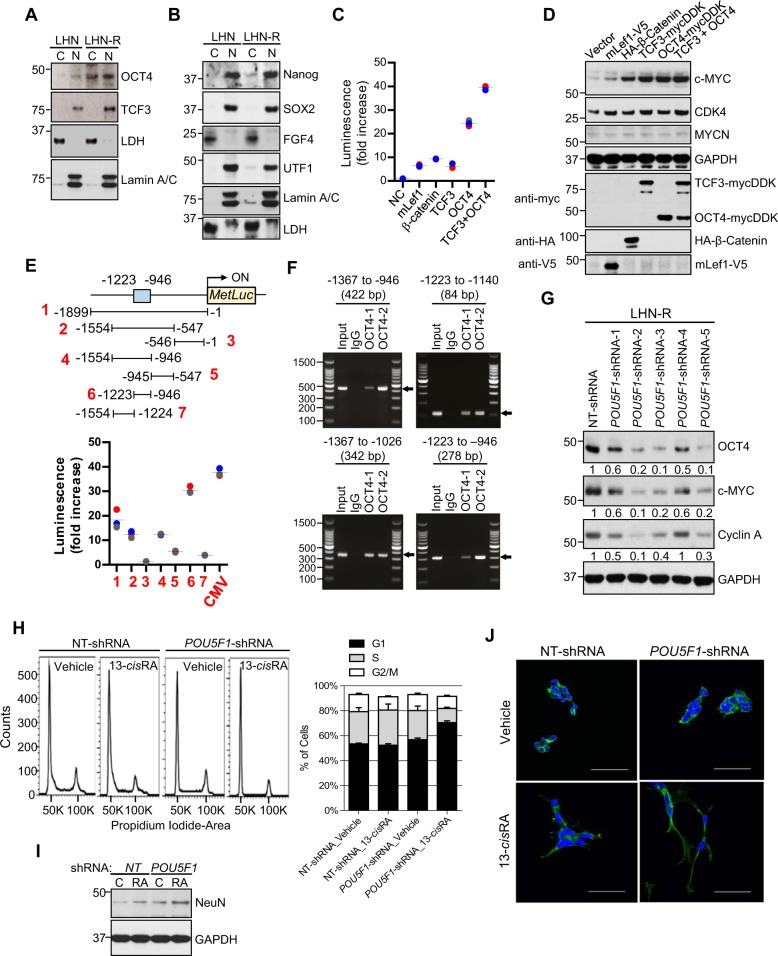
OCT4 and TCF3 regulate *MYC* transcription in 13-*cis*RA-resistant LHN-R cells. **a** Confirmation of increased OCT4 and TCF3 proteins observed in a Combo protein/DNA array (Supplementary Fig. 2a) in LHN and LHN-R cells. The results were reproducible in a repeat experiment. **b** Expression of pluripotency factors (Nanog, SOX2, FGF4, UTF1) in subcellular fractions of LHN and LHN-R. The results were reproducible in a repeat experiment. **c** Transcriptional activation of *MYC* by OCT4 and TCF3. TCF3 and OCT4 evaluated in inducing *MYC* transcription activity using a MYC (−1/−1899) luciferase reporter gene assay in HEK293FT cells. Reporter gene and transcription factor constructs used are shown in Supplementary Fig. 3b. *mLef1* and *β-Catenin*: positive controls for MYC transcriptional activation. Symbols: data, Bars: mean. **d** Increased c-MYC protein by transiently co-transfecting the transcription factors in **c** (*OCT4, TCF3, OCT4*+*TCF3, or positive controls)* into HEK293FT. The results were reproducible in a repeat experiment. *mLef1* and *β-Catenin*: positive controls. **e** Determination of OCT4-binding sites in *MYC* promoter region. Top: *MetLuc* reporter constructs with a series of *MYC* genomic DNA fragments. Bottom: the *MetLuc* reporter constructs (4 μg) *MYC*^*−1/−1899*^, *MYC*^*−547/−1554*^, *MYC*^*−1/−546*^, *MYC*^−^^*946/−1554*^, *MYC*^*−547/−945*^, *MYC*^*−946/−1223*^, and *MYC*^*−1224/−1554*^ were co-transfected with *pCMV6-POU5F1-mycDDK* (4 μg) into HEK293FT cells and *MetLuc* enzymatic activity was measured. *CMV* immediate early promoter (pCMV IE): a positive control. Symbols: data, Bars: mean. **f** OCT4 binding to proximal enhancer region of *MYC* confirmed by ChIP in LHN-R. Left: Locations of the four sets of amplicons (**a**–**d**) used to detect ChIP-enriched DNA fragments in the *MYC* enhancer region shown relative to the transcription start site (arrow). Right: detection of enriched fragments from ChIP of the four amplicons using ChIP-grade OCT4-1 and OCT4-2 antibodies or normal rabbit IgG antibody. **g** c-MYC and cyclin A protein levels in stable clones of LHN-R cells transduced with shRNA targeting *POU5F1* (*POU5F1*-shRNA-1 to -5) relative to non-targeting shRNA (NT-shRNA). **h** Cell cycle analyses of LHN-R cells stably transduced with either NT-shRNA or *POU5F1*-shRNA-2, followed by vehicle control or 13-*cis*RA for 14 days (NT-shRNA: *p* = 0.5, POU5F1: *p* ≤ 0.01). Left: representative histograms, right: percentage of cells in each phase of cell cycle. **i** NeuN protein expression in LHN-R cells stably transduced with either NT-shRNA or *POU5F1*-shRNA-2, and treated with either vehicle control (Veh) or 13-*cis*RA (RA) for 14 days. **j** Neurite outgrowth in LHN-R cells stably transduced with either NT-shRNA or *POU5F1*-shRNA-2, followed by vehicle control or 5 μM 13-*cis*RA for 14 days. Confocal microscopy (Blue: DAPI, Green: Phalloidin). Scale bar: 50 μM.

Having demonstrated that the deletion of *MYC* restored sensitivity to 13-*cis*RA, we sought to identify transcription factors (TF) that bind to the MYC gene promoter/enhancer region and drive *MYC* overexpression using a Combo Protein/DNA Array of 345 specific TF DNA-binding sequences (Supplementary Fig. 2a). The TFs with >2-fold increase or >50% reduction in LHN-R relative to LHN are depicted in Supplementary Fig. 2b, c. Of the TFs increased, two stemness markers, TCF3 (encoded by the *TCF3* gene)^20^ and OCT4 (encoded by the *POU5F1* gene)^21^ were noted. Both mRNA and protein expression of TCF3 and OCT4 were higher in LHN-R relative to LHN cells (Fig. 2a and Supplementary Fig. 2c); this was not seen for other stemness factors (Fig. 2b). To demonstrate that OCT4 and TCF3 drives *MYC* activation in neuroblastoma, expression of *POU5F1* (encoding OCT4) was transiently knocked down using siRNA in LHN-R cells. As anticipated, *POU5F1* or *TCF3* knockdown reduced c-MYC protein expression in LHN-R cells (Supplementary Fig. 3a). Activation of *MYC* gene transcription by OCT4 and/or TCF3 was determined by a luciferase reporter gene assay using a 1.9-kb genomic fragment of the *MYC* promoter/enhancer cloned from LHN-R cells (Supplementary Fig. 3b). The *MYC* reporter gene showed significant activation by TCF3 (6.2-fold), OCT4 (24.4-fold), and TCF3 + OCT4 (39.5-fold) compared with vector control (Fig. 2c). Transfection of the indicated constructs showed that TCF3 and OCT4 increased endogenous c-MYC protein and its downstream target CDK4 while MYCN levels were not affected (Fig. 2d). These data demonstrate that OCT4 and TCF3 individually and cooperatively regulate *MYC* transcription although MYC protein was not further increased by the combination of OCT4 and TCF3.

We next identified OCT4-binding sites in the *MYC* promoter/enhancer region. Transfac®, a database of TF’s and their DNA-binding sites, identified several potential OCT4-binding sites from −1 base to −1.9 kb of the promoter/enhancer region of *MYC*. Using various constructs (Fig. 2e), we confirmed binding of OCT4 to the region between −946 and −1223 of the *MYC* promoter/enhancer (Fig. 2e). OCT4 binding was further narrowed down to a 70-bp region located between −1209 and −1140 using a *MYC* reporter gene. Within the 70-bp sequence, two OCT4-binding regions were identified from −1204 to −1182 (OBS1) and from −1159 to −1145 (OBS2). To corroborate the direct binding of OCT4 to the binding sites within the *MYC* proximal enhancer region, ChIP experiments were conducted using two ChIP-grade OCT4 antibodies and nuclear extracts of LHN-R. Four different amplicons (84–422 bp) spanning the −1209/−1140 region of *MYC* promoter were confirmed to have ChIP-enriched OCT4-binding (Fig. 2f). The direct binding of OCT4 to the enhancer of *MYC* was also verified by electrophoretic mobility shift assay (EMSA), using a biotin-conjugated 44-bp (OBS1; MYC^−^^1209/−1166^), 29-bp (OBS2; MYC^−^^1173/−1145^), or 70-bp double-stranded DNA probe (OBS1 + OBS2; MYC^−1209/−1140^) (Supplementary Fig. 3c). These demonstrated that OCT4 binds to the *cis*-regulatory sequences in the *MYC* enhancer in 13-*cis*RA-resistant neuroblastoma cells.

### OCT4-induced 13-*cis*RA resistance mediated by *MYC* transcriptional activation

In colon cancer, TCF3 has been reported to induce *MYC* transcriptional activation, but we found that OCT4 has a greater role in *MYC* transcription than TCF3 (Fig. 2c). Thus, we focused on OCT4 and generated a stable clone of LHN-R with *POU5F1* knockdown by transducing five different short hairpin RNA sequences (*POU5F1*-shRNA-1 to -5) to evaluate the effect of OCT4 on 13-*cis*RA resistance in neuroblastoma cells. *POU5F1* knockdown decreased c-MYC and cyclin A protein expression in LHN-R cells (Fig. 2g) relative to a non-targeted control shRNA (NT-shRNA). Although NT-shRNA-transduced LHN-R cells did not show changes in S-phase DNA content when treated with 13-*cis*RA for 14 days (26 ± 3% vs 28 ± 5%, *p* = 0.58), the cell cycle progression of *POU5F1* knockdown cells was significantly reduced by 13-*cis*RA (23 ± 4% vs 11 ± 1%, *p* < 0.01) (Fig. 2h). Moreover, increased NeuN expression, a mature neuronal marker (Fig. 2i) and neurite outgrowth (Fig. 2j) and were observed in *POU5F1*-shRNA-transduced cells treated with 13-*cis*RA. These results suggest that a reduction in OCT4 restores 13-*cis*RA sensitivity to 13-*cis*RA-resistant neuroblastoma.

### POUs domain of OCT4 is required for *MYC* activation

Of the three variants of OCT4^21^, the POU domain is highly conserved through evolution^22^ and is a bipartite DNA-binding unit composed of two DNA-binding domains, POU-specific domain (POUs) and POU homeodomain (POU_HD_), tethered by a flexible linker^21^. Residues on POU_HD_ activates downstream target gene transcription^23^. To demonstrate that binding of OCT4 protein in the *MYC* enhancer region activated *MYC* transcription, a series of deletion mutants of OCT4 (Supplementary Fig. 4a) were co-expressed with a *MYC* reporter gene. Loss of the POUs domain (OCT4^Δ138–212^) resulted in a failure of OCT4 binding to the *MYC*^*−1209/−1140*^ enhancer and subsequent abrogation of *DDK-MYC-ER* expression (Supplementary Fig. 4b, lane 10). However, the OCT4 deletion mutant of residues 231 to 289 (OCT4^Δ231–289^) in the POU_HD_ domain interacted with the *MYC*^*−1209/*^^*−*^^*1140*^ enhancer to drive c-MYC expression (Supplementary Fig. 4b, lane 11), indicating OCT4 POU_HD_ is not necessary for *MYC* transcriptional activation. Truncated proteins lacking the NTD were able to interact with the *MYC*^−^^*1209/−1140*^ enhancer but lost their abilities to activate *MYC* expression (Supplementary Fig. 4b, lanes 7–9). Taken together, the NTD and POUs domains of OCT4 were indispensable for binding to the *MYC*^−*1209/−1140*^ enhancer and activating its gene expression.

### OCT4/MYC activation by MAPKAPK2 (MK2) caused 13-*cis*RA resistance and MK2 expression was associated with poor clinical outcome

**Fig. 3:**
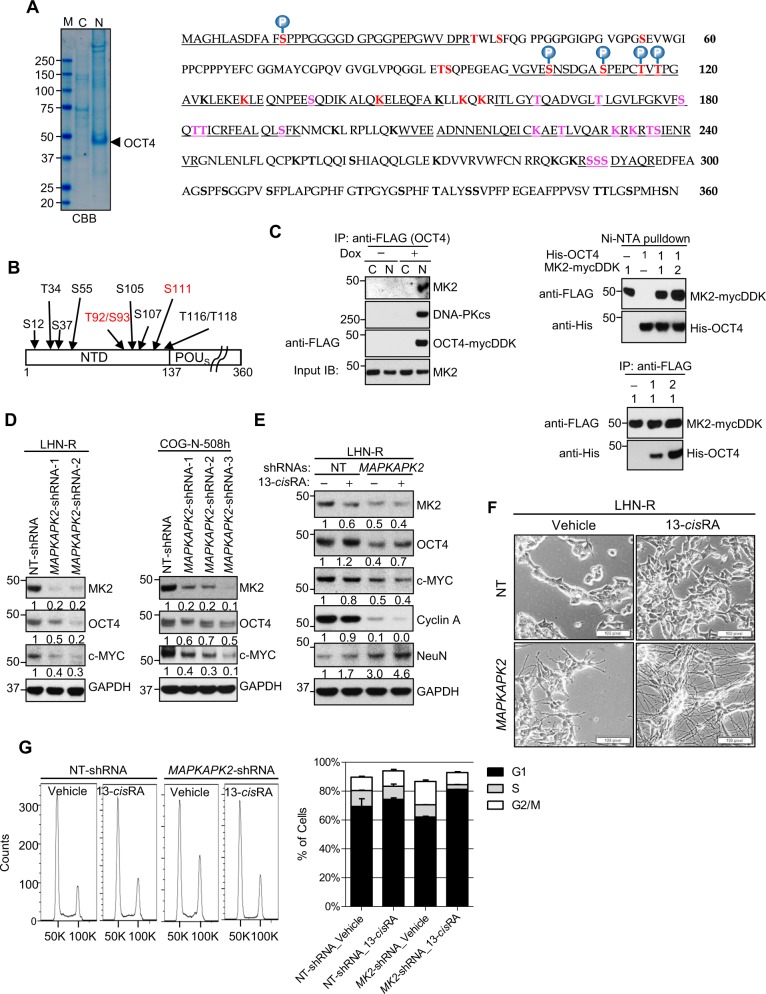
OCT4 interacting proteins by protein identification (ID) and post-translational modification (PTM) and verification of the protein expression and the role of the interacting MK2 protein in 13-*cis*RA resistance. **a** Left: Coomassie brilliant blue stained SDS-PAGE gel separating immunoprecipitated subcellular fractions of cells with exogenous OCT4 expression using anti-FLAG antibody. M: Marker, C: cytosolic, N: nuclear. For protein ID, the entire lanes were analyzed, and for PTM, the specific OCT4 band was subjected to mass spectrometry analyses. Right: OCT4 protein sequence with PTM status identified by mass spectrometry. Underline: mass spectrometry coverage, purple: not phosphorylated or acetylated, Red: phosphorylated, acetylated, or undetermined. **b** Predicted PTM sites in NTD and POUs domains of OCT4 interacting with/phosphorylated by MK2 (S^111^) and DNA-PKcs (S^93^). **c** Left: Direct nuclear interaction of MK2 or DNA-PKcs with OCT4 confirmed by immunoprecipitation in LHN-R cells stably transduced with the doxycycline-inducible construct of wild-type OCT4 with mycDDK-tag. Right: The direct interaction between OCT4 and MK2 is also confirmed by Ni-NTA pull down (top panel), and also by immunoprecipitation by FLAG using purified proteins (bottom panel). **d** Effect of stable *MAPKAPK2* knockdown on OCT4 and c-MYC protein expression in LHN-R (selected for resistance to 13-*cis*RA in the laboratory) and COG-N-508h (established from a PD patient sample after 13-*cis*RA treatment). The results were reproducible in a repeat experiment. **e** Protein expression of OCT4, MK2, Cyclin A, and NeuN (mature neuronal marker) in LHN-R cells with *MAPKAPK2* knockdown. Cells stably transduced with non-targeting NT-shRNA or *MAPKAPK2*-shRNA were treated with vehicle control or 5 μM 13-*cis*RA for 14 days. The results were reproducible in a repeat experiment. **f** Reversal of 13-*cis*RA resistance shown as neurite outgrowth in *MAPKAPK2* knockdown LHN-R cells. Cells stably transduced with non-targeting NT-shRNA or *MAPKAPK2*-shRNA were treated with vehicle control or 5 μM 13-*cis*RA for 14 days. A scale bar: 100 μM. **g** Reversal of 13-*cis*RA resistance shown as cell cycle arrest in *MAPKAPK2* knockdown LHN-R cells. Cells stably transduced with non-targeting NT-shRNA (S-phase cells: 11 ± 0.5% vs 9 ± 2.2%, *p* = 0.11) or *MAPKAPK2*-shRNA (S-phase cells: 8.5 ± 0.1% vs 3.2 ± 0.2%, *p* < 0.01) were treated with vehicle control or 13-*cis*RA for 14 days. Left: representative histograms, right: percentage of cells in each phase of cell cycle.

Mass spectrometry analyses identified OCT4-binding proteins and also determined post-translational modification of OCT4 in LHN-R cells transduced with mycDDK-tagged *POU5F1* (Fig. 3a). The mass spectrometry and the prediction of kinase interactions with OCT4 generated from PhosphoMotif Finder (Supplementary Table 3) identified two kinases, DNA-PKcs and MAPKAPK2 (MK2), which bind OCT4 and phosphorylate at S93 (DNA-PKcs) and S111 (MK2) (Fig. 3b), and this nuclear binding was confirmed by subcellular fractionation co-IP (Fig. 3c, left). The direct binding of MK2 and OCT4 was verified using purified proteins by Ni-NTA pull down (Fig. 3c, top) or anti-FLAG IP (Fig. 3c, bottom).

MK2 is a downstream substrate of p38MAPK pathway and post-transcriptinally regulates cytokines, indicating its role as a pro-inflammatory mediator^24^. By regulating phosphorylation and mRNA stability, MK2 also involves in actin remodeling, cell migration, cell cycle, and apoptosis^25–27^. To determine the role of MK2 in OCT4 activation, *MAPKAPK2* (gene encoding MK2) was stably knocked down in several neuroblastoma cell lines, including COG-N-508h a cell line established from a patient that progressed after 13-*cis*RA treatment. *MAPKAPK2* knockdown resulted in reduced OCT4 and c-MYC (Fig. 3d), and the *MAPKAPK2* knockdown cells treated with 13-*cis*RA showed reduced Cyclin A, increased expression of NeuN (Fig. 3e), increased neurite outgrowth (Fig. 3f), and arrested cell cycle (Fig. 3g), indicating the restoration of sensitivity to 13-*cis*RA. Neurite outgrowth in response to 13-*cis*RA in *MAPKAPK2* knockdown cells was also seen in the PD neuroblastoma cell line COG-N-443h (Supplementary Fig. 5a, b).

**Fig. 4:**
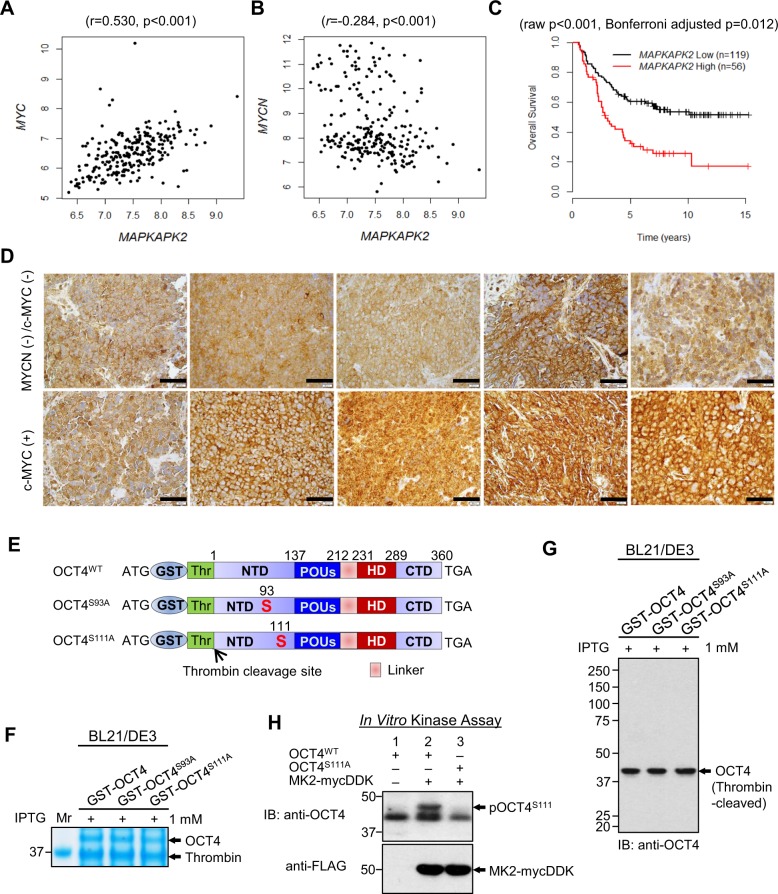
MK2 as a biomarker for 13-*cis*RA resistance and prognosis in neuroblastoma. **a** Positive correlation between mRNA expression of *MYC* and *MAPKAPK2* in 249 neuroblastoma patients. Expression data from the neuroblastoma NCI TARGET database (https://ocg.cancer.gov/programs/target/data-matrix). **b** Inverse correlation between *MYCN* and *MAPKAPK2* mRNA expression in 249 neuroblastoma patients (Data: NCI TARGET database https://ocg.cancer.gov/programs/target/data-matrix). **c** Overall survival of patients by *MAPKAPK2* mRNA expression in neuroblastoma from the NCI TARGET database. Of the total patients (*n* = 247), only patients with *MYCN* non-amplification (*n* = 175) were used for the analysis. The data was scanned to identify maximum separation of the curves, and the *p*-value was adjusted by Bonferroni adjustment. The overall survival using the median *MAPKAPK2* expression and the event-free survival are shown in Supplementary Fig. 5c–e. **d** MK2 protein expression by immunohistochemistry staining in neuroblastoma primary tumors collected at diagnosis with low expression of both MYCN and c-MYC proteins (upper panel) and with high c-MYC protein (lower panel) expression. A white scale bar: 20 μM, a black scale bar: 20 μM. **e** Constructs (plasmid: pGEX-4T-1) encoding a human wild-type OCT4 or OCT4 mutants (S93A and S111A) with a GST tag and thrombin cleavage site at the NH_2_-terminus. **f** Human recombinant OCT4 proteins expressed in BL21/DE3 strain of *E. coli* after IPTG induction, GST column purification, and thrombin cleavage subjected to SDS-PAGE and stained with Coomassie brilliant blue solution. **g** Proteins in **f** detected by immunoblotting using anti-OCT4 antibody. **h** In vitro kinase assay of MK2 on phosphorylating OCT4WT and OCT4^S111A^. The results were reproducible in a repeat experiment.

Given that MK2 is activated via phosphorylation, the correlation of mRNA or protein levels of MK2 may not indicate the activity of the enzyme. However, in a search of potential biomarkers in existing database, we evaluated the relationship of *MAPKAPK2, MYC*, or *MYCN* expression and patient survival in the NCI TARGET database of mRNA expression for 175 MYCN non-amplified primary neuroblastoma tumors^28^. *MAPKAPK2* expression strongly positively correlated with *MYC* expression, but inversely correlated with *MYCN* expression (Fig. 4a, b, *p* < 0.001). In addition, we observed that high *MAPKAPK2* expression was associated with poor survival (Fig. 4c, adjusted *p* = 0.012, Supplementary Fig. 5c–e). In neuroblastoma primary tumors with low MYCN, tumors with high c-MYC protein had a trend of higher MK2 protein expression relative to those with low MYCN and c-MYC proteins (Fig. 4d).

### Expression of pMK2 and pOCT4 activating c-MYC were associated with progressive disease in neuroblastoma

We next bacterially expressed the human recombinant proteins of wild-type OCT4 and OCT4 mutants (S93A and S111A, constructs shown in Fig. 4e, verified the expression in Supplementary Fig. 6a, b), and purified them followed by thrombin cleavage (Fig. 4f, g). An in vitro MK2 kinase assay using the recombinant proteins showed that MK2 phosphorylated OCT4^WT^ but not OCT4^S111A^ (Fig. 4h).

**Fig. 5:**
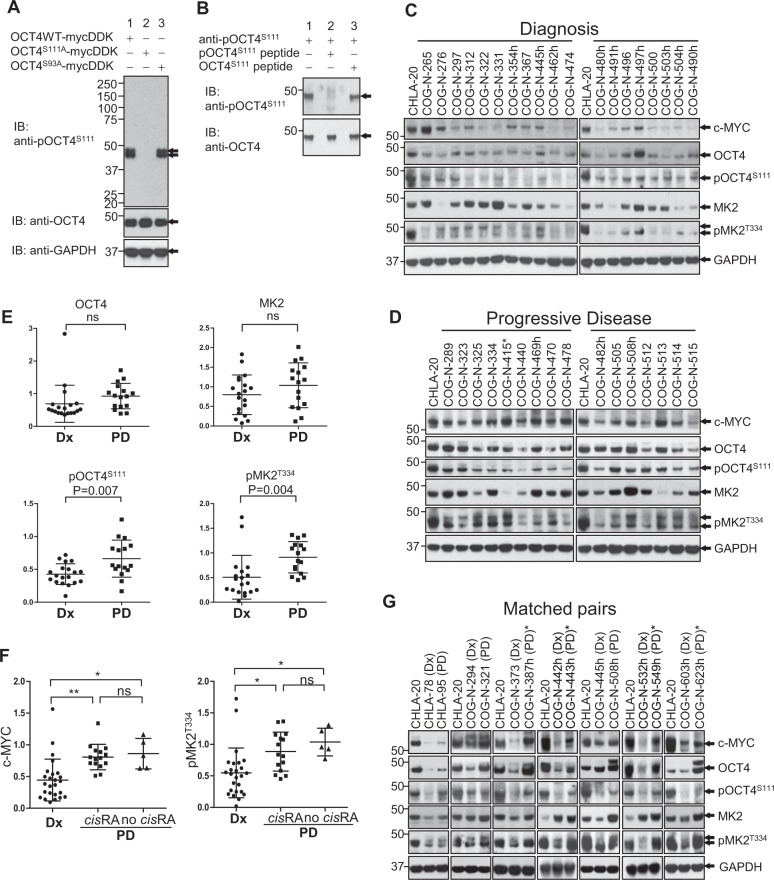
Higher pMK2 and pOCT4^S111^ in PD with high c-MYC. **a** Specificity of anti-pOCT4S111-antibody tested in HHN-R cells with exogenous expression of OCT4WT and OCT4^S111A^, and OCT4^S93A^. **b** Specificity of anti-pOCT4S111-antibody tested by neutralizing antibody with pOCT4^S111^ peptide or OCT4^S111^ peptide, and detecting pOCT4^S111^ in LHN-R cells, stably expressing OCT4WT. **c** c-MYC, OCT4, phosphoOCT4 (pOCT4^S111^), MK2, and phosphoMK2 (pMK2^T334^) expression in patient-derived neuroblastoma cell lines established from samples obtained pre-therapy at diagnosis from patients with high-risk neuroblastoma. CHLA-20: control for two different membranes. c-MYC and GAPDH protein levels are from Fig. 1a. **d** c-MYC, OCT4, phosphoOCT4 (pOCT4^S111^), MK2, and phosphoMK2 (pMK2^T334^) expression in patient-derived neuroblastoma cell lines established from progressive disease clinical samples. CHLA-20: control for two different membranes. * Patient not treated with 13-*cis*RA prior to obtaining sample. c-MYC and GAPDH protein levels are from Fig. 1b. **e** Dot plots quantitating immunoblotting data from **a** and **b**. The values were normalized in two ways by the expression of specific proteins in CHLA-20 and GAPDH. **f** Dot plots of c-MYC and pMK2 protein expression comparing cell lines established at Dx to cell lines established at PD from patients treated with 13-*cis*RA (*cis*RA), and at PD from patients not treated with 13-*cis*RA (no *cis*RA) cell lines. ** and *: significant, ns: not significant. **g** c-MYC, OCT4, phosphoOCT4 (pOCT4^S111^), MK2, and phosphoMK2 (pMK2^T334^) expression in matched pairs of cell lines established at diagnosis (Dx) and progressive disease (PD) from seven different neuroblastoma patients. CHLA-20 was used as the control for each membrane. CHLA-78 (Dx) and CHLA-95 (PD) is a pair established from patients before 13-*cis*RA became standard of care for high-risk neuroblastoma. * Patient not treated with 13-*cis*RA prior to obtaining sample.

To demonstrate that S111 residue of OCT4 is phosphorylated in cell lines established from PD patients as well as LHN-R cells, a custom antibody of pOCT4^S111^ was produced. The specificity was confirmed in LHN-R cells exogenously expressing OCT4^WT^ and two mutants. Anti-pOCT4^S111^ antibody did not recognize the S111A mutant OCT4 but it reacted with OCT4^WT^ and the S93A mutant while all three proteins were detected by anti-OCT4 antibody (Fig. 5a). Neutralized antibody (epitope saturation with an excess of pOCT4^S111^ peptide) did not detect pOCT4^S111^, whereas the antibody pre-incubated with OCT4^S111^ peptide was able to detect pOCT4^S111^ (Fig. 5b), confirming specificity of the anti-pOCT4^S111^ antibody.

The constitutive protein expression of c-MYC, OCT4, pOCT4^S111^, MK2, and pMK2 was compared between cell lines established pre-therapy (Dx) (Fig. 5c) and those established at time of progressive disease (PD) (Fig. 5d, quantitation data in Fig. 5e) in non-paired as well as paired (Dx and PD established from the same patient) cell lines (Fig. 5g). c-MYC protein levels in Fig. 5 are from Fig. 1a, b to show the association between c-MYC and OCT4/pOCT4^S111^/MK2/pMK2. The levels of c-MYC, pOCT4^S111^, and pMK2 were significantly higher in PD cell lines compared with Dx cell lines (*p* < 0.01) from other patients. OCT4 levels were not significantly different due to one outlier (COG-N-497h). In matched-pair cell lines (Dx and PD from same patient), the seven PD cell lines showed higher expression of c-MYC, OCT4, and pMK2 relative to autologous Dx lines from the same patients. We compared cell lines established from patients who progressed prior to 13-*cis*RA treatment to cell lines from patients treated with 13-*cis*RA (Fig. 5f). c-MYC and pMK2 were significantly higher in PD cell lines with no prior 13-*cis*RA exposure relative to Dx lines and no significant differences were seen between PD lines from patients treated with 13-*cis*RA and PD lines without 13-*cis*RA. Thus, pMK2-mediated increased c-MYC expression is not limited to tumors exposed to 13-*cis*RA.

**Fig. 6:**
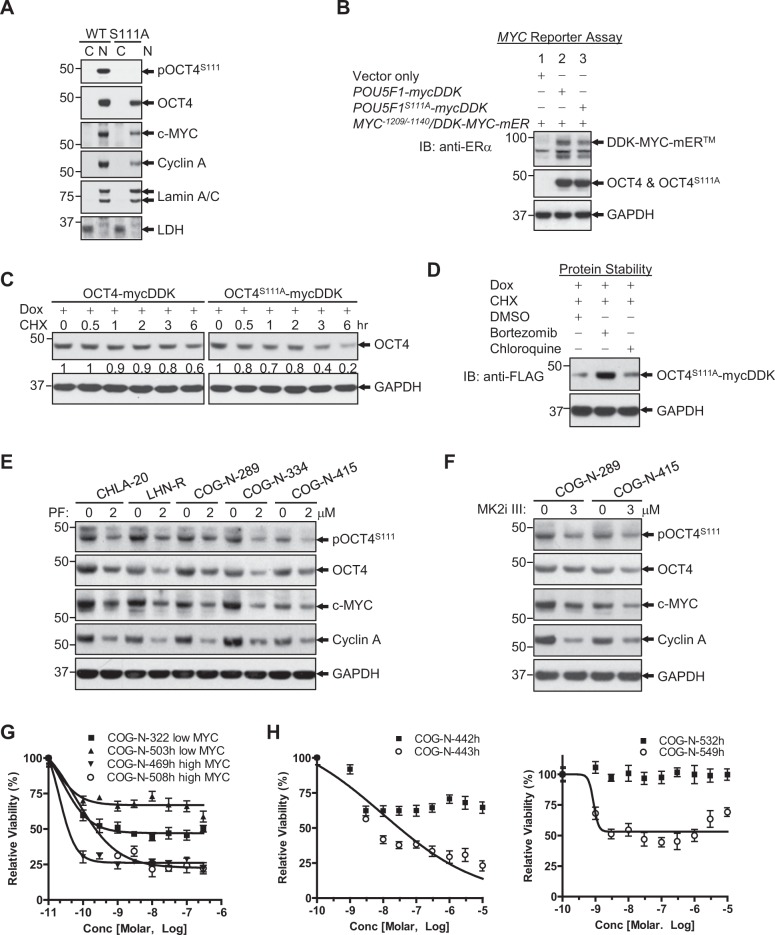
The role of MK2 phosphrylation at S111 in c-MYC increase and the effect of MK2 inhibition in cell viability. **a** OCT4 at S111 residue is phosphorylated in LHN-R. OCT4WT and OCT4^S111A^ mutant were exogenously expressed in LHN-R cells. The expression of pOCT4^S111^, OCT4, c-MYC, and Cyclin A was evaluated in subcellular fractions. **b** Assessment of DNA-binding ability of wild-type OCT4 and mutant OCT4^S111A^ using *MYC*^*−1209/−1140*^/*MYC* reporter assay system. Empty vector, *POU5F1-mycDDK* and *POU5F1*^*S111A*^*-mycDDK* (4 μg each) were separately co-transfected with reporter gene *MYC*^*−1209/−1140*^*/DDK-MYC-mER*^*TM*^ (4 μg) in HEK293FT cells. After 48 h, the equal amount of protein lysates (20 μg) were run and analyzed by SDS/PAGE and WB using specific antibodies, as indicated. Anti-ERα: DDK-c-MYC-mER^TM^ expression, anti-DDK (FLAG): expression of exogenous mycDDK-tagged wild-type OCT4 and its mutant. **c** OCT4 stability was decreased in OCT4^S111A^ mutant relatively to OCT4^WT^, shown by cycloheximide (CHX) treatment. The cells were transduced with a doxycycline-inducible system to exogenously express OCT4^WT^ or OCT4^S111A^ mutant, treated with doxycycline (Dox) for 48 h, followed by cycloheximide (CHX) incubation for various times before immunoblotting. **d** Rescue of OCT4^S111A^ protein degradation by bortezomib. The LHN-R cells were transduced with a Dox-inducible system to exogenously express OCT4^S111A^ mutant, treated with Dox for 24 h, followed by 10 μg/ml of cycloheximide (CHX) along with Bortezomib (1 μM, proteasome inhibitor) or Chloroquine (50 μM, lysosome inhibitor) incubation for 6 h before immunoblotting. DMSO was served as a vehicle control. **e** Decreased pOCT4^S111^, OCT4, c-MYC, and Cyclin A in CHLA-20 (PD cell line from patients not treated with 13-*cis*RA), LHN-R, COG-N-289, COG-N-334, and COG-N-415 (the last three PD cell lines were established from patients treated with 13-*cis*RA) treated with an MK2 inhibitor (PF3644022). **f** Decreased pOCT4^S111^, OCT4, c-MYC, and Cyclin A in COG-N-289 and COG-N-415 (the PD cell lines established from patients treated with 13-*cis*RA) treated with an MK2 inhibitor (MK2iIII). **g** Relative viability of neuroblastoma cells (two cell lines with high c-MYC: COG-N-469h and COG-N-508h, two cell lines with low c-MYC: COG-N-322 and COG-N-503h) treated with PF3644022 (0–300 nM) for 96 h in six replicates. **h** Relative viability of neuroblastoma cells (two sets of matched pairs: COG-N-442h and COG-N-443h, COG-N-532h, and COG-N-547h) treated with PF3644022 (0–10 μM) for 96 h in six replicates. The results were reproducible in a repeat experiment.

When the S111 residue of OCT4 was mutated, pOCT4^S111^ was not detected (Fig. 6a). S111 mutation did not affect the nuclear translocation of the protein, but it did result in decreased cyclin A (Fig. 6a), reduced c-MYC expression (Fig. 6b), and decreased OCT4 protein stability (half-lives of proteins: >6 vs 3 h, Fig. 6c). When we further treated these cells with chloroquine or bortezomib in the presence of cycloheximide (CHX), bortezomib was able to rescue OCT4^S111A^ from degradation (Fig. 6d), suggesting that the degradation of the mutated OCT4 is expedited via proteasomes. MK2 inhibition by a small molecule MK2 inhibitors resulted in decreased pOCT4^S111^, OCT4, c-MYC, and Cyclin A in CHLA-20, LHN-R, COG-N-289, COG-N-334, and COG-N-415 (Fig. 6e, f). In vitro cytotoxicity testing showed that cell lines with high c-MYC were more sensitive than low c-MYC lines to an MK2 inhibitor in four non-paired cell lines (Fig. 6g) and two pairs of matched-pair cell lines (Fig. 6h). These data suggest that the inhibition of OCT4 phosphorylation at S111 residue affects the stability of OCT4.

**Fig. 7:**
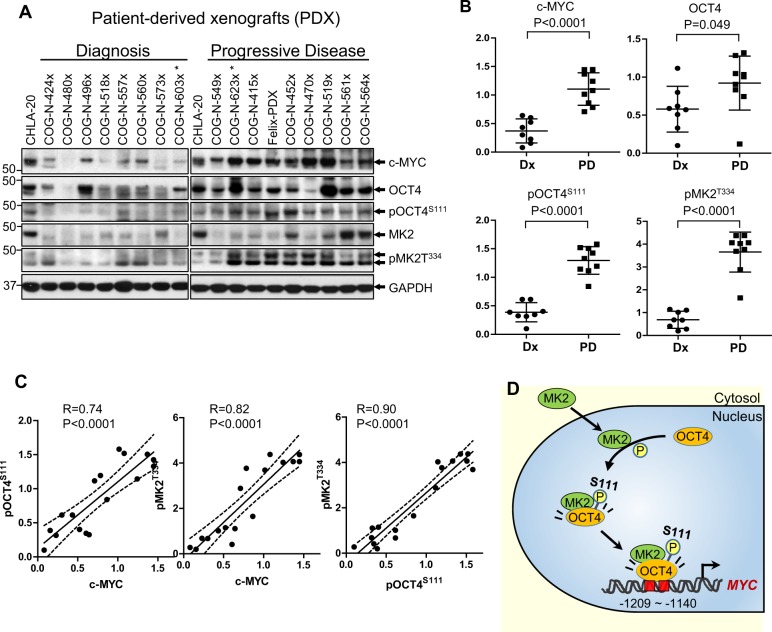
A novel transcriptional activation pathway of c-MYC by OCT4 phosphorylated by MK2. **a** c-MYC, OCT4, phosphoOCT4 (pOCT4^S111^), MK2, and phosphoMK2 (pMK2^T334^) expression in patient-derived xenografts (PDXs) of neuroblastoma established from clinical samples of obtained at diagnosis (*n* = 8) and at progressive disease (*n* = 9). CHLA-20 was used as the control for the two membranes. * COG-N-603x and COG-N-623x are matched Dx-PD pair of PDXs established from the same patients. The matched-pair direct-to-culture cell lines are COG-N-603h and COG-N-623h shown in Fig. 5g. **b** Dot plots quantitating immunoblotting data from **a**. The values were normalized in two ways by the expression of specific proteins in CHLA-20 and GAPDH. **c** Correlation between c-MYC and pOCT4^S111^, c-MYC and pMK^T334^, and pOCT4^S111^ and pMK^T334^. The results of linear regression analyses with 95% confidence interval (dotted line) are presented (*n* = 17). **d** Proposed mechanism of c-MYC transcriptional activation in progressive disease neuroblastoma. MK2 shuttles into the nuclei of cells and phosphorylates OCT4 at S111 residue. There are two OCT4-binding sites in the c-MYC promoter/enhancer region between −1209 and −1140. The binding of pOCT4^S111^ increases transcriptional activation of *MYC*.

Protein expression of c-MYC, pOCT4^S111^, and pMK2^T334^ was significantly higher in neuroblastoma PDXs (Supplementary Table 2) established at progressive disease compared with PDXs established at diagnosis (Fig. 7a, b). Although the PDX sample size was smaller than cell lines, a strong correlation was seen between c-MYC and pOCT4^S111^ as well as c-MYC and pMK2^T334^ (Fig. 7c). Also, expression of c-MYC, pOCT4^S111^, and pMK2^T334^ from pre-therapy was higher than progressive disease in a matched pair of Dx/PD cell lines (603 h/623 h, Fig. 5g) and the corresponding PDXs (603×/623×) established from the same patient (Fig. 7a). We assessed phosphorylation of both T334 and T222 (Figs. 5 c, d, g, 7a, Supplementary Fig. 7a, b) as both are required for maximal activation of MK2^29,30^. Thus, phosphorylation of OCT4 by MK2 transcriptionally activates *MYC* and this oncogenic pathway is upregulated in progressive disease relative to pre-therapy neuroblastomas.

## Discussion

*MYCN* genomic amplification is well established as one of the prognostic markers in neuroblastoma^31,32^. More recently, the prognostic significance of c-MYC has been reported in a small group^33^, and then in a large cohort of neuroblastoma patients with undifferentiated/poorly differentiated tumors^18^. Although *MYC* amplification is rare in neuroblastoma, high c-MYC protein in patients with undifferentiated tumors was as frequent as 11% at diagnosis, suggesting that *MYC* transcriptional activation rather than gene amplification is a driver of aggressive tumor behavior in most c-MYC-driven neuroblastomas. As tumor biopsies of patients at PD are infrequently obtained from children with neuroblastoma, we have instead utilized low-passage cell lines and PDXs established under uniform conditions from bone marrow or blood of children with neuroblastoma at Dx prior to therapy and also at PD. Our discovery of c-MYC mediating resistance to 13-*cis*RA and being highly expressed in PD neuroblastoma suggests that *MYC* transcriptional activation occurs both in a subset of patients at diagnosis and likely more frequently in tumors at time of PD.

Tumors that recurred after *PIK3CA* inactivation acquired focal amplification of *MET* or *MY*C in an in vivo mouse breast cancer model expressing *PIK3CA*^*H1047R*^^34^. Also, the MYC/eIF4E axis is a mediator of drug resistance to BEZ-235 in breast cancer cells^35^. In leukemia, *MYC* activation compromised tyrosine kinase inhibitor sensitivity and functional inhibition of *MYC* overcame resistance by promoting differentiation^36^. In our experiments, *MYCN* regulation by 13-*cis*RA was not attenuated by repeated exposure to the drug in neuroblastoma cells, but escape from sustained suppression of *MYCN* occurred via *MYC* transcriptional activation. *MYC* and *MYCN* are structurally and functionally homologous^37^, and *Mycn* can functionally replace *Myc* in murine development, cell growth and differentiation^38^. We showed that *MYC* knockdown increased MYCN protein, but the cells did not regain their proliferative properties, suggesting that 13-*cis*RA-resistant cells are addicted to c-MYC, which functionally replaces MYCN in 13-*cis*RA-resistant neuroblastoma. Four of the seven matched-pair cell line and xenograft models were established from patients who were not treated with 13-*cis*RA due to rapid progression of the disease and the PD cell lines from four pre/post therapy pairs along with one non-paired PD cell line showed elevated pMK2 and c-MYC. Thus, the OCT4/c-MYC axis we discovered likely is a mechanism of early disease progression in some patients and is acquired to promote resistance to 13-*cis*RA in others.

In the current study, we defined MK2 phosphorylation of OCT4 as a novel mechanism for oncogenic activation of *MYC* in neuroblastoma (Fig. 7d). Oct4 is an important transcription factor that controls self-renewal and pluripotency in the early stages of mammalian embryogenesis^39^, and it cannot be replaced by any other POU family members in reprogramming somatic cells into induced pluripotent stem cells (iPSC)^40^. Ectopic expression of OCT4 blocks progenitor-cell differentiation and causes dysplasia in epithelial tissues^41^. Not only is OCT4 required to maintain the pluripotency of human embryonic stem cells, but it is also involved in lineage specification^42,43^. For reprogramming somatic cells, OCT4 along with c-MYC, KLF4, and SOX2 are required to induce pluripotency in both human and mouse somatic cells in vitro^44^. Other studies demonstrated that higher expression of OCT4 occurs in embryonic or cancer stem cells^39,45–47^. One study showed *Oct4* is upstream of *mych*, a *Myc* family member, in the development of zebrafish^48^. Our study is the first to show OCT4 as an upstream regulator of c-MYC in human cancer. Previous studies in embryonic stem cells showed that phosphorylation sites in OCT4 POU_HD_ (T235, S236, and S229) are important for pluripotency^49,50^. This is especially important as our data suggest that the function of OCT4 in cancer may be distinguished from its role in pluripotency. The downstream activation of genes by OCT4, in cancer or in pluripotency will depend on kinase-specific phosphorylation of residues.

Our data establish the MK2/OCT4/c-MYC axis as a mechanism of progressive disease neuroblastoma regardless of prior to 13-*cis*RA exposure. This mechanism of *MYC* transcriptional activation may be operative in other cancer types and could potentially be an important therapeutic target. The p38 MAPK (p38) kinase, upstream of MK2, has been investigated as a therapeutic target in inflammatory diseases due to its role in the regulation of the mRNA stability of TNF-α, along with several other mediators with unknown immune responses^51^. Many p38 inhibitors were tested in clinical trials but none progressed to phase III mainly due to systemic toxicities (hepato-, cardiac toxicities, and CNS disorders)^52,53^, possibly due to p38 being involved in the regulation of more than 60 substrates with various physiological roles^54^. For that reason, MK2 (the first substrate of p38) is being tested as an alternative target to p38 in inflammatory diseases^55^. Regardless, experiments were conducted to confirm p38 as the upstream activator of MK2 in neuroblastoma (Supplementary Fig. 7c). As our data show that MK2 mediates c-MYC activation, which is an important oncogenic pathway in many cancers, and direct inhibition of c-MYC is challenging^56^, inhibiting MK2 action on OCT4 is potentially a novel therapeutic target in cancers with high c-MYC expression.

It has been reported that the degradation of retinoid receptors is known to induce resistance to retinoids. However, in LHN cells and its resistant clone, LHN-R, no differences in the expression of RAR-β was observed (Supplementary Fig. 7d), indicating that the degradation of retinoic receptors is not the mechanism of resistance to 13-*cis*RA in neuroblastoma.

In summary, our studies define the MK2/OCT4/c-MYC axis as a novel mechanism of *MYC* transcriptional activation in neuroblastoma that mediates resistance to 13-*cis*RA by rescuing 13-*cis*RA induced MYCN downregulation and is commonly found in progressive disease neuroblastomas. Regulation by MK2 of OCT4 binding to newly identified binding sites in the promoter/enhancer region of *MYC* may provide a novel molecular target for regulating the *MYC* oncogene. This novel mechanism for regulation of c-MYC expression warrants investigation in cancers other than neuroblastoma.

## Methods

### Materials and reagents

Chloroquine diphosphate (CQ), cycloheximide (CHX), isotretinoin (13-*cis*RA), NaF, NaHCO_3_, Na_3_VO_4_, Tris-HCl, Triton X-100, Aprotinin, Leupeptin, Pepstatin A, PMSF, Ethanol (molecular biology), Isopropanol (molecular biology), ITS, Puromycin, and 3× FLAG peptide were from Sigma-Aldrich; DTT, EDTA, Formaldehyde (molecular biology), Glycine (molecular biology), IPTG, NaCl, MES, SDS, TAE, Tween-20, FBS, DMEM, IMDM, RPMI-1640, l-Glutamine, Pen Strep, Sodium pyruvate, Trypsin/EDTA, Lipofectamine®, PLUS^TM^ reagent, Proteinase K, RNase A, and Superscript® III First-Strand Synthesis System from ThermoFisher Scientific; Tet-free FBS and Doxycycline from Clontech; Ni-NTA from EMD Millipore; NH_2_-terminal His-tagged human recombinant OCT4 protein (purity > 90%, made in *E. coli*) from ProteinONE; COOH-terminal mycDDK-tagged human recombinant MK2 protein from OriGene; MK2 inhibitor PF3644022 from Tocris; MK2 inhibitor III from Santa Cruz Biotechnoloyg; bortezomib from LC Laboratories; p38 inhibitor SB203580 from Sellectchem; *Age1-HF*, *BamH1-HF*, *EcoR1-HF*, *Mlu1-HF*, *Not1-HF*, *Pme1*, *Sgf1*, *Xba1*, and *Xho1* restriction enzymes from New England Biolabs; bovine serum albumin from Jackson ImmunoResearch Laboratories; All oligonucleotides were synthesized from Integrated DNA Technologies (IDT).

### Collections of human neuroblastoma clinical samples and their cell lines and derivatives

All clinical specimens from patients ≥18 months of age with stage 4 neuroblastoma at diagnosis (Dx) and post therapy progressive diseases (PD) were collected from year 2000 through 2016 at the Cancer Center laboratory of C. Patrick Reynolds in the Texas Tech University Health Sciences Center (TTUHSC, Lubbock, TX). The Children’s Oncology Group (COG) provides aliquots of these samples to the laboratory of Dr. Reynolds for the specific purposes of patient-derived cell line and xenograft establishment (The COG repository for cell lines and xenografts: www.CCcells.org). For the proposed activities, enrollment of specimen donors was performed by a strict procedure that has been approved by the Institutional Review Boards at participating institutions, with informed consent and the utmost attention to the issues of patient safety, anonymity and confidentiality. In neuroblastoma patients, tumor samples from PD are rarer than Dx samples as tumor re-biopsy at relapse is often not clinically indicated. In contrast, bone marrows and blood samples are often obtained from neuroblastoma patients at relapse. Cell lines established from tumors collected at Dx and at PD and cell lines with matching Dx/PD pairs (established from seven patients) were cultured in IMDM medium supplemented with 2 mM l-Glutamine, human 1× ITS [10 µg/ml Insulin, 5.5 µg/ml Transferrin (iron-free), and 5 ng/ml Sodium selenite], and 20% heat-inactivated FBS. They were mycoplasma-free and routinely checked for cell line identify using short tandem repeat genotyping as compared with the original primary sample material within the database: www.CCcells.org. The models used are show in Supplementary Tables 1, 2.

### Establishment of 13-*cis*RA-resistant neuroblastoma models

To provide models for defining the molecular mechanisms of resistance to 13-*cis*RA, SMS-LHN (LHN) human neuroblastoma cell line (MYCN expressing but lacking *MYCN* or *MYC* genomic amplification)^17^ was treated with a clinically achievable concentration of 13-*cis*RA (5 μM) according to the dosing schedule clinically used in high-risk patients (i.e., 2-week on/2-week off/6 cycles). The 13-*cis*RA resistance was considered established when neurite outgrowth was not seen in the presence of 13-*cis*RA, and the resistant variant was named SMS-LHN-R (LHN-R). The cells were maintained in RPMI-1640 supplemented with 10% heat-inactivated FBS.

### Immunoblotting and immunoprecipitation

The cell lines and seven matched pairs of Dx/PD human neuroblastoma samples were prepared for IP or IB as described previously^57^. Densitometric analysis of protein quantitation was determined by Quantitiy One® 1D software v4.6.

### RT-PCR

Total RNAs were extracted from LHN and LHN-R cells by RNeasy Mini Kit (Qiagen) as per the instructions. TaqMan qPCR primers used in the study are: *MYC*, Hs02602824_cn (amplicon length 87 bp); *MYCN*, Hs02718426_cn (amplicon length 81 bp); and GAPDH (Hs.PT.39a.22214836, Integrated DNA Technologies). The total RNAs (100 ng) were mixed with TagMan One-Step RT-PCR Master Mix Reagents (Applied Biosystems) in an ABI MicroAmp optical 96-well plate. The analysis of mRNA expression was performed with qPCR using ABI QuantStudio^TM^ 12 K Flex system. Below are the RT-PCR thermal cycling conditions: RT reaction for 30 min at 48 °C, hold for 10 min at 95 °C, and then PCR 15 s at 95 °C and 1 minute at 60 °C for 45 cycles.

### *MYC* gene knockout by CRISPR/Cas9

The LHN-R cells were seeded overnight in a 6-well culture plate with complete RPMI-1640/10% FBS. The Plasmid DNA solution (150 μl), including *MYC* CRISPR/Cas9 KO plasmid (h) and *MYC* HDR plasmid (1 μg each), was added directly to the dilute UltraCruz® Transfection Reagent (150 μl), and incubate for 20 min at room temperature. The *MYC* CRISPR/Cas9 KO plasmid (h) consists of a pool of three plasmids, each encoding the Cas9 and a target-specific 20-nucleotide gRNA designed for *MYC* knockout. When cells reach about 70–80% confluency, the Plasmid DNA/UltraCruz® Transfection Reagent Complex was added dropwise and gently mixed. After 96 h incubation, the cells were visually confirmed by detection of the RFP via fluorescent microscopy. Furthermore, the cells with *MYC* KO (+/− or −/−) were enriched by selection with complete medium containing Puromycin (0.5 μg/ml) for at least 2–3 weeks. The *MYC* knockout efficiency in the stable cell clones was confirmed by IB.

### Protein/DNA arrays hybridization and analysis

Panomics Protein/DNA Combo Array carrying 345 specific DNA-binding elements was used for identification of TFs critical for gene regulation in 13-*cis*RA sensitive cells LHN and the resistant cells LHN-R, according to the instruction manual (Affymetrix). The signals were densitometrically quantified and normalized to an internal standard using Image Studio^TM^ software v5.x.

### Cloning and mutagenesis

The mER^TM^ cDNA was amplified by the Expand High Fidelity PCR System using *pBABE-MYC*^*439*^*-mER*^*TM*^ (kind gift of Dr. Trevor Littlewood, Department of Biochemistry, University of Cambridge, UK) as a template. The PCR-amplified *mER*^*TM*^ gene was subcloned into the *Mlu*1/*Pme*1 sites of *pCMV6-MYC*^*454*^*-mycDDK* (CUG start codon; full-length human MYC^454^ cDNA with encoding 454 amino acids) to create *pCMV6-MYC*^*454*^*-mER*^*TM*^ using LigaFast^TM^ Rapid DNA Ligation System. The DDK (=FLAG) tag was added to the NH_2_ terminus of *pCMV6-MYC*^*454*^*-mER*^*TM*^ using *Eco*R1 and *Sgf*1 sites (*pCMV6-DDK-MYC*^*454*^*-mER*^*TM*^). The wild-type *MYC*^*439*^ (AUG start codon; 439 amino acids) and *MYC*^*454*^ mutant forms, including *MYC*^*454Δ121-158*^ (same with *MYC*^*439Δ106-143*^)*, MYC*^*454Δ72-209*^ (same with *MYC*^*439Δ57-194*^), and *MYC*^*454V409D*^ (same with *MYC*^*439V394D*^), were sequentially inserted in-frame into the *Sgf*1/*Mlu*1 sites of *pCMV6-DDK-MYC*^*454*^*-mER*^*TM*^ to replace the wild-type *MYC*^*454*^. The *DDK-mER*^*TM*^ (vector control), *DDK-MYC*^*439*^*-mER*^*TM*^, *DDK-MYC*^*454*^*-mER*^*TM*^, *DDK-MYC*^*454Δ121-158*^*-mER*^*TM*^ (TAD deletion), *DDK-MYC*^*454Δ72-209*^*-mER*^*TM*^ (TAD plus MBIII deletions), and *DDK-MYC*^*454V409D*^*-mER*^*TM*^ (V409, a site for the interaction of MYC with MIZ-1) DNA fragments were also ligated into the *EcoR1*/*Pme1* sites of lentiviral vector *pLenti-c-mycDDK-IRES-Puro*, respectively. The genes encoding mLef1 and β-Catenin were reverse transcribed and PCR-amplified from CD-1 mouse skin tissue and human cervical cancer cell line HeLa, and then the blunt-end cDNA was directly cloned into *pcDNA3.1D/V5-His-TOPO*^*C*^ and *pCMV6-AN-HA* vectors. The c-MYC and OCT4 mutant clones of deletions and point substitutions were generated with specific primers using QuikChange Site-Directed Mutagenesis Kit. DNA sequences of all constructs were verified by using an automated ABI-3730xl DNA Analyzer and ABI PRISM^®^ BigDye^TM^ Terminator v3.0 Ready Reaction Cycle Sequencing Kit in MacroGen USA (Maryland). All plasmid DNAs were prepared using purification kits from Qiagen and were endotoxin-free.

### Luciferase reporter assay

Luciferase reporter assay was performed using Ready-To-Glow^TM^ Secreted Luciferase Reporter System according to manufacturer’s instructions (Clontech). In addition, a secreted alkaline phosphatase (SEAP) reporter plasmid was included in all transfections for normalization. The relative fold increase in luciferase activity was normalized with the SEAP activity.

### Construction of regulatory sequences of *MYCN*^promoter^ and *MYC*^enhancer^ into the *DDK-MYC-ER* gene reporter system

The *DDK-MYC*^*454*^*-mER*^*TM*^ DNA fragment was amplified and inserted into the *Age*1/*Not*1 sites of pMetLuc2 Reporter to replace *Luc* gene (henceforth *DDK-MYC-ER*). The double-stranded (ds) DNA fragments for WT/*MYC*^−1209/−1176^ (*Eco*R1/*Bam*H1), MT3/*MYC*^−1209/−1140^ (*Xho*1/*Bam*H1), and MT4/*MYC*^−1209/−1140^ (*Xho*1/*Bam*H1) were formed by heating forward/reverse primers at 95 °C for 5 min and re-annealing at 37 °C for 1 h. The WT/*MYC*^−1209/−1176^, MT3/*MYC*^−1209/−1140^ and MT4/*MYC*^−1209/^^−^^1140^ were ligated separately with the *Eco*R1/*Age*1, *EcoR*1/*Age*1, *Eco*R1/*Bam*H1, *Xho*1/*Bam*H1, and *Xho*1/*Bam*H1 sites of the *DDK-MYC-ER* reporter. *MYCN*^promoter^ (*Xho*1/*Bam*H1) (−1/−1098 upstream of the AUG start site of *MYCN*) was amplified with its specific primers using SK-N-BE(2) genomic DNA as a template. PCR products were cut with *Xba*1/*Bam*H1*-HF*, *EcoR1-HF*/*BamH1-HF* and *Xho1*/*BamH1-HF*, respectively, and then subcloned into *DDK-MYC-ER* or *MYCN-mycDDK* reporter. 293FT cells were co-transfected with these (4 µg each) along with mock vector (*pCMV6-entry-mycDDK*) or vector expressing *POU5F1* and the indicated mutant forms (4 µg each) for 48 h. The whole-cell lysates were subjected to SDS-PAGE/IB, and then probed with the indicated antibodies. The primers used in the reaction are shown in Supplementary Table 4.

### Electrophoretic mobility shift assay (EMSA)

EMSA^58^ was conducted using the Panomics’ EMSA Gel Shift Kit (Affymetrix) according to the manufactures’ instructions. The sequences of biotin-labeled double-stranded WT *MYC* probes are shown in Supplementary Table 4.

### Chromatin immunoprecipitation (ChIP) assay

ChIP analysis was performed as previously described^59^. Briefly, the proteins in LHN-R-resistant neuroblastoma cells were cross-linked to DNAs with 1% formaldehyde for 10 min at room temperature. The reaction for protein/DNA cross-linking was stopped by addition of Glycine (125 mM). Cells were rinsed twice with ice-cold 1× PBS and harvested by a silicone scraper. After centrifugation for 5 min at 1000×*g*, cells were lysed subsequently with the ice-cold lysis buffer (iL1b and iL2). Cells were then re-suspended in complete shearing buffer iS1b with protease inhibitors to shear cross-linked protein/DNA to an average size of 200–1000 bp by sonication using UCD-300 Bioruptor Plus (Diagenode). An aliquot of the sheared chromatin DNAs were used as an input. The chromatin extracts containing protein/DNA fragments were ChIPed at 4 °C overnight using ChIP-grade rabbit anti-OCT4-1 or rabbit anti-OCT4-2 polyclonal antibody mixing together with DiaMag protein A-coated magnetic beads. The normal rabbit IgG and CTCF antibody were used as negative and positive control, respectively. The magnetic beads were washed four times with wash buffer W1, W2, W3, and W4, and DNA fragments were eluted with elution buffer iE1 and decross-linked with iE2. After 4 h incubation at 65 °C, DNA was washed with Wash buffer 1 and 2 and then enriched by purification using IPure beads. The semi-quantitative real-time PCR was performed using the Mastercycler (Eppendorf). PCR parameters used in the reaction were as follows: 95 °C/3 min; 40 cycles with denaturation at 95 °C/20 s, annealing at 60 °C/20 s, and polymerization at 72°C/20 s. PCR products were analyzed electrophoretically on 2% agarose gels and amplification was performed in triplicate and repeated twice. Relative occupancy values were calculated by determining the apparent ChIP efficiency (ratios of the amount of immunoprecipitated DNA to that of the input sample) and normalized to the level observed at a control region, which was defined as 1.0. The four sets of primers used to quantify the DNA fragments of *MYC* from the ChIP-enriched DNA are listed in Supplementary Table 4.

### RNAi-mediated gene silencing

Transfection of mammalian cells was carried out as described in the Amaxa’s cell line database (Lonza). Briefly, LHN-R-resistant cells were electroporated with 50 or 100 nM of scramble non-targeting, *POU5F1* (71264) or *TCF3* (s13874) pre-designed siRNA (Ambion) in solution SF using program EN-150 of the Amaxa 4D-Nucleofector^TM^ System (Lonza). Cells were grown in RPMI-1640/10% FBS complete medium for 2–3 days at 37 °C in a 5% CO_2_ incubator. The protein levels of OCT4 and TCF3 were validated using IB with specific antibodies.

### Construction of shRNAs of *POU5F1* and *MK2*

Four human *POU5F1* shRNAs (*POU5F1*-shRNA-1, -3, -4 and -5) and *MK2* shRNAs (1, 2, and 3) in *pLKO.1-puro* lentiviral vector designed by The RNAi Consortium at the Broad Institute of MIT were purchased from Dharmacon. *POU5F1* shRNAs include shRNA-1/TRCN0000004879; shRNA-3/TRCN0000004880; shRNA-4/TRCN0000004881; and shRNA-5/TRCN0000004882. Meanwhile, the pre-designed siRNA for *POU5F1* (71264; Ambion), as validated above, was used as a *POU5F1*-shRNA-2 targeting sequence. These short hairpin oligonucleotides with sense and antisense strands were mixed together, denatured at 95 °C for 5 min and re-annealed at 37 °C for 1 h to form a duplex DNA. *POU5F1*-shRNA-2 insert was then ligated into *pLKO.1-puro* lentiviral vector with *puro* antibiotic marker for selection of stable clones in mammalian cells. The *pLKO.1-puro eGFP* shRNA sequence, which targets eGFP, was used as a non-targeting control (NT-shRNA). *MK2* shRNA antisense sequences were also purchased from Dharmacon (shRNA-1/TRCN0000002282; shRNA-2/TRCN0000002283; and shRNA-3/TRCN0000002284).

### Cell transduction

293FT cells (Invitrogen) were cultured in DMEM/10% FBS supplemented with 2 mM Glutamine, 100 U/ml Pen Strep and 1 mM Sodium pyruvate. 293FT cells were plated on a 10-cm tissue culture dish and incubated at 37 °C 5% CO_2_ incubator until they reach 70–80% confluency. 293FT cells were co-transfected either lentiviral ORFs or shRNAs (5 µg each) along with Lenti-vpak Packaging Kit using MegaTran 1.0 Transfection Reagent. After 72 h transfection, the virus-containing medium was collected, spun down, filtered (0.45 µm) and used for targeting into the cells of interest (LHN and LHN-R) by infection. The stable clones were obtained after 2–3 weeks of selection in RPMI-1640/10% FBS with Puromycin (0.5 µg/ml).

### Assay of 13-*cis*RA resistance by flow cytometric analysis of nuclear DNA contents

Effect of 13-*cis*RA treatment on cell proliferation was determined by examining the distribution of cells in different phases of the cell cycle by flow cytometry. The LHN, LHN-R, LHN-*MYC* stable clones, and LHN-R-*POU5F1* knockdown clones were separately plated in a T75 flask overnight. The cells for testing 13-*cis*RA resistance were treated with vehicle or 13-*cis*RA (5 µM) in the complete medium for 14 days with the drugs being replenished every 96 h. To evaluate the cell cycle arrest, cells (10^6^) were washed once with ice-cold 1× PBS and then fixed and permeabilized in 70% ethanol at −20 °C for 1 h. After centrifugation, cells were incubated with 0.5 ml PI/RNase staining buffer (BD Biosciences) in the dark at room temperature. After 15 min, DNA content was analyzed using a LSR II flow cytometer (BD Biosciences), which was operated with DiVa software v4.1.2. DNA content histogram analysis was performed using FlowJo software v7.6.

### Neurite outgrowth by confocal microscopy

LHN-R-NT-shRNA and LHN-R-*POU5F1*-shRNA-2 cells were treated with vehicle or 13-*cis*RA for 14 days. Neurite outgrowth was observed using Alexa Fluor 488 phalloidin under confocal microscope, as previously described^60^.

### Protein identification by MALDI-TOF and post-translational modification by Triple Quad mass spectrometry

OCT4-mycDDK protein was pulled down by IP using EZview Red anti-FLAG affinity purification, and the protein was separated on SDS-PAGE, stained with the Colloidal blue, and cut out for analysis at the Proteomics Core Facility of University of Texas Southwestern Medical Center, Dallas, TX.

### Immunohistochemistry

Immunohistochemistry staining (IHC) for MK2 protein was performed as described earlier^18^. Five samples each for c-MYC(+) and c-MYC(−)/MYCN(−) were selected for MK2 staining by IHC.

### Purification of human recombinant wild-type OCT4 and its mutant proteins

The BL21-DE3 (EMD Millipore) was transformed by heat-shock method with the pGEX-4T1 plasmid encoding a human wild-type OCT4 or OCT4 mutants (S93A and S111A) with a GST tag and a thrombin recognition sequence at the NH_2_-terminus. The expression of GST-OCT4, GST-OCT4^S93A^, and GST-OCT^S111A^ recombinant proteins were induced by adding IPTG (1 mM) for 3 h at 37 °C with a vigorous shaking until the cell density reads at OD_600_ = 0.6–0.8. The *E. coli* cells were harvested by spinning at 4000 × *g* for 20 min at 4 °C and then re-suspended in BugBuster® Master Mix (EMD Millipore) with complete EDTA-free protease and phosphatase inhibitors (Roche). After incubation the bacterial suspensions were completely lysed on a rotating mixer for 20 min at room temperature, and the crude extracts were centrifuged at 16,000×*g* for 20 min at 4 °C. The soluble extracts were loaded directly onto the GST SpinTrap^TM^ columns with Glutathione Sepharose^TM^ 4B (GE Healthcare), which were pre-equilibrated by adding 1× PBS binding buffer (pH = 7.4). The GST-tagged proteins were bound to the Glutathione Sepharose^TM^ 4B. After washing three times, the bound GST-OCT4 or its mutant fusion proteins were cleaved by an in-gel digestion with thrombin protease (GE Healthcare) for 16 h at 22 °C, followed by removal of the GST tag by centrifugation. The thrombin-cleaved OCT4, OCT4^S93A^, and OCT4^S111A^ recombinant proteins were concentrated by Amicon Ultra Centrifugal Filters with M.W. 10 kDa cut-off (EMD Millipore), and the protein concentration was determined by BCA assay (Pierce). The purified recombinant proteins were subjected to SDS-PAGE and confirmed by staining with Colloidal blue solution (Invitrogen).

### In vitro MK2 kinase assays

For MK2 kinase assays, the purified MK2-mycDDK (1 μg) was incubated at 37 °C for 30 min in 1x kinase buffer [containing Tris-HCl (pH = 7.5; 25 mM), β-Glycerophosphate (5 mM), MgCl_2_ (10 mM), DTT (2 mM), Na_3_VO_4_ (0.1 mM), and ATP (200 μM)] plus wild-type OCT4 or mutant OCT4^S111A^ (1 μg each). The reactions were quenched by the addition of 4× LDS loading buffer and 100 mM DTT, followed by boiling at 95 °C for 10 min. The protein samples were resolved on SDS-PAGE and IB with anti-OCT4 antibody.

### Custom anti-human phosphoOCT4^S111^ rabbit polyclonal antibody

Anti-phosphoOCT4^S111^ antibody (RRID_AB_2721810) was produced by GenScript. Anti-pOCT4^S111^ antibody was prepared by immunizing two New Zealand rabbits four times with a NH_2_-terminal KLH-conjugated phosphopeptide SNSDGA{pS}^57^ PEPCTVT as an antigen. The phospho-specific antibody was affinity-purified through a phosphopeptide-conjugated Sepharose CL-4B column. Eluted IgG was then passed through the corresponding nonphosphorylated peptide (SNSDGASPEPCTVT) column to deplete any IgG that were not specific to pOCT4^S111^.

### Patient-derived xenograft (PDX) models

The 6-to-8-week-old nu/nu mice (Envigo) were subcutaneously injected with patient-derived xenografts at 10–20 million cells (100 μl) per mouse. Tumor volume was measured by ½ length × width × height. Mice were sacrificed once the tumor volume exceeded 500 mm^3^ for the purpose of our experiments. The animal protocol to establish PDX models is reviewed and approved by the TTUHSC Institutional Animal Care and Use Committee (IACUC).

### Statistical analysis

Student’s *t*-test was used to determine statistically significant differences on MS Excel. *p*-values were two-sided and tests were considered significant at *p* < 0.05. All the experiments were performed in triplicate and were consistently repeatable; for simplicity, one representative experiment for each condition is shown.

NCI TARGET data: Correlation between *MYCN*, *MYC*, and *MK2* were assessed numerically and graphically. Specifically, scatter plots were generated to demonstrate the relationships between these genes, and the corresponding Spearman correlations were calculated. Overall survival was defined as the time elapsed from study enrollment to death, with those living at the time of last follow-up censored. Median expression of *MK2* was used to categorize patients into low and high expression groups. Log-rank test was used for comparing overall survival of these two groups, for *MYCN* non-amplified patients (*n* = 175) of all patients (*n* = 247). Indeed, *MK2* expression cut-off that achieved the best separation of patient survival was obtained by scanning the whole expression range, with a Bonferroni adjustment of *p*-value performed. All analyses were performed by using the SAS software (Windows v9.4).

## Supplementary information

Suppl Table 1

Suppl Table 2

Suppl Table 3

Suppl Table 4

Suppl Figure Legends

Suppl Figure 1

Suppl Figure 2

Suppl Figure 3

Suppl Figure 4

Suppl Figure 5

Suppl Figure 6

Suppl Figure 7
