# Supplementary material for: MYC transcription activation mediated by OCT4 as a mechanism of resistance to 13-cisRA-mediated differentiation in neuroblastoma
Source: Cell Death Dis. 2020 May 14;11(5):368. doi: 10.1038/s41419-020-2563-4 (PMC7224192; doi:10.1038/s41419-020-2563-4)
Supplement: Supplementary file 1 — Suppl Table 1 [file 41419_2020_2563_MOESM1_ESM.docx]

**Supplementary Table 1.** Clinical annotations of tumor samples from which cell lines were established.

| **Cell Line** | **Phase of Therapy** | **Sample Type** | **INSS stage** | ***MYCN*** | ***MYC*** | **Ploidy** | **LOH** | **Histology** | **Age at Diagnosis** | **Primary site** | **13-*cis*RA treatment** |  |
| --- | --- | --- | --- | --- | --- | --- | --- | --- | --- | --- | --- | --- |
| **Diagnosis Cell Lines** | | | | | | | | | | | | |
| COG-N-265 | Dx | tumor | 4 | N | N | 1 | 1 | 2 | 4.06 | Left adrenal gland | No |  |
| COG-N-276 | Dx | bone marrow | 4 | A | N | 1 | *NA* | *NA* | 6.49 | Left adrenal gland | No |  |
| COG-N-297 | Dx | tumor | 4 | N | N | *NA* | *NA* | 2 | 2.47 | Adrenal gland, NOS Suprarenal gland Adrenal, NOS | No |  |
| COG-N-312 | Dx | bone marrow | 4 | N | N | 1 | *NA* | 2 | 1.49 | Peripheral nerves and Autonomic nervous system of abdomen P | No |  |
| COG-N-322 | Dx | tumor | 4 | N | N | 1 | *NA* | 2 | 1.19 | Adrenal gland, NOS Suprarenal gland Adrenal, NOS | No |  |
| COG-N-331 | Dx | bone marrow | 4 | N | N | 2 | *NA* | 2 | 9.37 | Adrenal gland, NOS Suprarenal gland Adrenal, NOS | No |  |
| COG-N-354h | Dx | bone marrow | 4 | N | N | 2 | *NA* | 2 | 1.05 | Adrenal gland, NOS Suprarenal gland Adrenal, NOS | No |  |
| COG-N-367 | Dx | bone marrow | 4 | N | N | 1 | *NA* | 2 | 1.99 | Adrenal gland, NOS Suprarenal gland Adrenal, NOS | No |  |
| COG-N-445h | Dx | bone marrow | 4 | N | N | 1 | 1 | 2 | 1.85 | Retroperitoneum Periadrenal tissue Perinephric tissue Per | No |  |
| COG-N-462h | Dx | bone marrow (right draw) | 4 | N | N | 1 | *NA* | *NA* | 2.04 | Adrenal gland, NOS Suprarenal gland Adrenal, NOS | No |  |
| COG-N-474 | Dx | bone marrow | 4 | N | N | 1 | 0 | *NA* | 2.39 | Retroperitoneum Periadrenal tissue Perinephric tissue Per | No |  |
| COG-N-480h | Dx | bone marrow (right draw) | 4 | N | N | 1 | 0 | 2 | 0.95 | Adrenal gland, NOS Suprarenal gland Adrenal, NOS | No |  |
| COG-N-490h | Dx | bone marrow | 4 | N | N | 2 | 1 | 2 | 1.90 | Retroperitoneum Periadrenal tissue Perinephric tissue Per | No |  |
| COG-N-491h | Dx | peripheral blood | 4 | N | N | 2 | 1 | 2 | 1.90 | Retroperitoneum Periadrenal tissue Perinephric tissue Per | No |  |
| COG-N-496 | Dx | bone marrow | 4 | N | N | *NA* | 0 | 2 | 3.20 | Abdomen, NOS Abdominal wall, NOS Intra-abdominal site, NOS | No |  |
| COG-N-497h | Dx | bone marrow  (left draw) | 4 | N | N | *NA* | *NA* | 2 | 1.62 | Adrenal gland, NOS Suprarenal gland Adrenal, NOS | No |  |
| COG-N-500 | Dx | bone marrow (right draw) | 4 | A | N | 2 | *NA* | 2 | 4.54 | Retroperitoneum Periadrenal tissue Perinephric tissue Per | No |  |
| COG-N-503h | Dx | bone marrow | 4 | A | N | 2 | 0 | 2 | 5.63 | Kidney, NOS Renal, NOS Kidney parenchyma | No |  |
| COG-N-504h | Dx | bone marrow  (left draw) | 4 | A | N | 2 | *NA* | 2 | 4.54 | Retroperitoneum Periadrenal tissue Perinephric tissue Per | No |  |
| **Progressive Disease Cell Lines** | | | | | | | | | | | | |
| COG-N-289 | PD | tumor | 4 | A | *N* | *NA* | *NA* | *NA* | 4.66 | Adrenal gland | Yes |  |
| COG-N-323 | PD | tumor | 4 | A | N | 1 | 0 | 2 | 15.19 | Peripheral nerves and Autonomic nervous system of head, face | Yes |  |
| COG-N-325 | PD | tumor | 4 | N | N | 1 | *NA* | 2 | 0.99 | Abdomen, NOS Abdominal wall, NOS Intra-abdominal site, NOS | Yes |  |
| COG-N-334 | PD | bone marrow | 4 | N | N | 2 | *NA* | 2 | 1.70 | Retroperitoneum Periadrenal tissue Perinephric tissue Per | Yes |  |
| COG-N-415 | PD | peripheral blood | 4 | A | N | 1 | 0 | 2 | 1.42 | Adrenal gland, NOS Suprarenal gland Adrenal, NOS | No* |  |
| COG-N-440 | PD-PM | blood | 4 | A | N | 1 | 1 | 1 | 0.93 | Adrenal gland, NOS Suprarenal gland Adrenal, NOS | Yes |  |
| COG-N-469h^∏^ | PD | bone marrow  (left draw) | 4 | N | N | *NA* | *NA* | 2 | 1.58 | Adrenal gland, NOS Suprarenal gland Adrenal, NOS | Yes |  |
| COG-N-470^∏^ | PD-PM | blood (autopsy) | 4 | N | N | *NA* | *NA* | 2 | 1.58 | Adrenal gland, NOS Suprarenal gland Adrenal, NOS | Yes |  |
| COG-N-478 | PD | bone marrow  (left draw) | 4 | N | N | *NA* | *NA* | 2 | 2.23 | Adrenal gland, NOS Suprarenal gland Adrenal, NOS | Yes |  |
| COG-N-482h | PD | bone marrow | 4 | N | N | 2 | *NA* | 2 | 3.70 | Abdomen, NOS Abdominal wall, NOS Intra-abdominal site, NOS | Yes |  |
| COG-N-505 | PD-PM | blood (autopsy) | 4S | N | N | 2 | 1 | 2 | 0.52 | Liver Hepatic, NOS | Yes |  |
| COG-N-508h | PD | bone marrow  (left draw) | 4 | A | N | 1 | 1 | 2 | 1.85 | Retroperitoneum Periadrenal tissue Perinephric tissue Per | Yes |  |
| COG-N-512# | PD | bone marrow  (left draw) | 4 | N | N | 2 | 0 | 2 | 3.04 | Adrenal gland, NOS Suprarenal gland Adrenal, NOS | Yes |  |
| COG-N-513¶ | PD | bone marrow (right draw) | 4 | N | N | 1 | 1 | 2 | 2.08 | Adrenal gland, NOS Suprarenal gland Adrenal, NOS | Yes |  |
| COG-N-514¶ | PD | bone marrow  (left draw) | 4 | N | N | 1 | 1 | 2 | 761 | Adrenal gland, NOS Suprarenal gland Adrenal, NOS | Yes |  |
| COG-N-515# | PD | bone marrow (right draw) | 4 | N | N | 2 | 0 | 2 | 1111 | Adrenal gland, NOS Suprarenal gland Adrenal, NOS | Yes |  |
| **Paired Cell Lines** | | | | | | | | | | | | |
| COG-N-294/  COG-N-321 | Dx/  PD | tumor/bone marrow | 3 | N | N | 1 | *NA* | 2 | 1.54 | Abdomen, NOS Abdominal wall, NOS Intra-abdominal site, NOS | Yes |  |
| COG-N-373/  COG-N-387h | Dx/  PD | bone marow | 4 | A | N | 1 | *NA* | *NA* | 3.73 | Adrenal gland, NOS Suprarenal gland Adrenal, NOS | No* |  |
| COG-N-445h/  COG-N -508h | Dx/  PD | bone marrow/  bone marrow  (left draw) | 4 | N | N | 1 | 1 | 2 | 1.85 | Retroperitoneum Periadrenal tissue Perinephric tissue Per | Yes |  |
| COG-N-442h/  COG-N -443h | Dx/  PD | bone marrow (left)/  bone marrow (right) | 4 | A | N | 1 | 1 | 1 | 1.40 | Retroperitoneum Periadrenal tissue Perinephric tissue Per | No* |  |
| COG-N-532h/  COG-N -549h | Dx/  PD | bone marrow | 4 | A | N | 2 | 0 | 2 | 1.23 | Abdomen, NOS Abdominal wall, NOS Intra-abdominal site, NOS | No* |  |
| COG-N-603h/ COG-N -623h | Dx/  PD | tumor | 4 | A | N | 1 | *NA* | 2 | 0.73 | Adrenal gland, NOS | No* |  |

*MYCN*: A, amplified; N, non-amplified.

LOH (loss of heterozygosity): 0, no; 1, yes

Histology: 1, favorable; 2, unfavorable

Ploidy: o, hypoploid; 1, diploid; 2, hyperploid

Age at diagnosis: in years

Dx: diagnosis, PD: progressive disease, PM: post-mortem

Unknown: information could not be verified

*Due to the time from diagnosis to progressive disease being short, the patients from which these cell lines were established from did not receive 13-cisRA.

∏, ¶, # Established from same patient at different times and different sites.
