# Supplementary material for: MYC transcription activation mediated by OCT4 as a mechanism of resistance to 13-cisRA-mediated differentiation in neuroblastoma
Source: Cell Death Dis. 2020 May 14;11(5):368. doi: 10.1038/s41419-020-2563-4 (PMC7224192; doi:10.1038/s41419-020-2563-4)
Supplement: Supplementary file 2 — Suppl Table 2 [file 41419_2020_2563_MOESM2_ESM.docx]

**Supplementary Table 2.** Clinical annotations of tumor samples from which patient-derived xenografts (PDXs) were established.

| **Cell Line** | **Phase of Therapy** | **Sample Type** | **INSS stage** | ***MYCN*** | ***MYC*** | **Injection**  **type** | **Histology** | **Age at Diagnosis** | **Primary site** | **13-*cis*RA treatment** |
| --- | --- | --- | --- | --- | --- | --- | --- | --- | --- | --- |
| **Diagnosis PDXs** | | | | | | | | | | |
| COG-N-424x | Dx | tumor | 4 | A | N | SQ | C | 0.5 | Adrenal gland, NOS Suprarenal gland Adrenal, NOS | No |
| COG-N-480x | Dx | bone marrow | 4 | A | N | SQ & TV | C | 0.95 | Adrenal gland, NOS Suprarenal gland Adrenal, NOS | No |
| COG-N-496x | Dx | bone marrow | 4 | A | N | SQ | C | 3.2 | N/A | No |
| COG-N-518x | Dx | bone marrow | 4 | A | N | SQ | C | 3.3 |  | No |
| COG-N-557x | Dx | tumor | 4 | A | N | SQ | C | 2.68 | Abdomen, NOS Abdominal wall, NOS Intra-abdominal site, NOS | No |
| COG-N-560x | Dx | tumor | 4 | A | N | SQ & TV | C | 1 | Abdomen, NOS Abdominal wall, NOS Intra-abdominal site, NOS | No |
| COG-N-573x | Dx | bone marrow | 4 | A | N | SQ | C | 2.31 | Suprarenal gland | No |
| COG-N-603x | Dx | tumor | 4 | A | N | SQ | C | 0.72 | Adrenal gland, NOS | No |
| **Progressive disease PDXs** | | | | | | | | | | |
| COG-N-549x | PD | tumor | 4 | A | N | SQ | C | 1.2 | Abdomen, NOS Abdominal wall, NOS Intra-abdominal site, NOS | No |
| COG-N-623x | PD | tumor | 4 | A | N | SQ | C | 0.72 | Renal, NOS | No |
| COG-N-415x | PD-PM | blood | 4 | A | N | SQ | C | 1.4 | Adrenal gland, NOS Suprarenal gland Adrenal, NOS | No |
| Felix-PDX | PD-PM | blood | 4 | A | N | SQ | C | 4 |  | Yes |
| COG-N-452x | PD-PM | blood | 4 | A | N | SQ | C | 1.4 | Adrenal gland, NOS Suprarenal gland Adrenal, NOS | Yes |
| COG-N-470x | PD-PM | blood | 4 | N | N | SQ | C | 1.58 | Adrenal gland, NOS Suprarenal gland Adrenal, NOS | Yes |
| COG-N-519x | PD-PM | blood | 4 | A | N | SQ | C | 2 | Adrenal gland, NOS | unknown |
| COG-N-561x | PD-PM | blood | 4 | A | N | SQ | C | 1.6 | Retroperitoneum | unknown |
| COG-N-564x | PD-PM | bone marrow | 4 | A | N | SQ | C | Unknown | unknown | unknown |

*MYCN*: A, amplified; N, non-amplified.

Age at diagnosis: in years

Dx: diagnosis, PD: progressive disease, PM: post-mortem

Unknown: information could not be verified

COG-N-603x and COG-N-623x were established from the same patient from tumor samples at diagnosis and at tumor at progressive disease post-chemotherapy.
