# Supplementary material for: MYC transcription activation mediated by OCT4 as a mechanism of resistance to 13-cisRA-mediated differentiation in neuroblastoma
Source: Cell Death Dis. 2020 May 14;11(5):368. doi: 10.1038/s41419-020-2563-4 (PMC7224192; doi:10.1038/s41419-020-2563-4)
Supplement: Supplementary file 3 — Suppl Table 3 [file 41419_2020_2563_MOESM3_ESM.docx]

**Supplementary Table 3.** Kinase/phosphatase substrate and binding motifs of OCT4 that are curated from the published literature.

| **Position in OCT4** | **Sequence in OCT4** | **Corresponding motif described in the literature (phosphorylated residues in red)** | **Features of motif described in the literature** |  |
| --- | --- | --- | --- | --- |
| 10 - 13 | AFSP | XXpSP | GSK-3, ERK1, ERK2, CDK5 substrate motif |  |
| 11 - 13 | FSP | X[pS/pT]P | GSK-3, ERK1, ERK2, CDK5 substrate motif |  |
| 12 - 13 | SP | pSP | ERK1, ERK2 Kinase substrate motif |  |
| 31 - 34 | DPRT | [E/D]XX[pS/pT] | Casein Kinase I substrate motif |  |
| 34 - 37 | TWLS | [pS/pT]XX[S/T] | Casein Kinase I substrate motif |  |
| 34 - 37 | TWLS | [pS/pT]XX[E/D/**pS*/pY***] | Casein Kinase II substrate motif |  |
| 36 - 41 | LSFQGP | X[pS/pT]XXX[A/P/S/T] | G protein-coupled receptor kinase 1 substrate motif |  |
| 53 - 56 | PGSE | XX[pS/pT]E | G protein-coupled receptor kinase 1 substrate motif |  |
| 91 - 95 | ETSQP | [E/D][pS/pT]XXX | β-Adrenergic Receptor kinase substrate motif |  |
| 92 - 94 | TSQ | XpSQ | DNA dependent Protein kinase substrate motif |  |
| 104 - 107 | ESNS | [E/D]XX[pS/pT] | Casein Kinase I substrate motif |  |
| 104 - 108 | ESNSD | [E/D][pS/pT]XXX | β-Adrenergic Receptor kinase substrate motif |  |
| 104 - 110 | ESNSDGA | XpSXXDXX | Pyruvate dehydrogenase kinase substrate motif |  |
| 105 - 107 | SNS | pSX[E/**pS*/pT***] | Casein Kinase II substrate motif |  |
| 105 - 108 | SNSD | pSXX[E/D] | Casein kinase II substrate motif |  |
| 105 - 108 | SNSD | [pS/pT]XX[E/D] | Casein Kinase II substrate motif |  |
| 105 - 108 | SNSD | [pS/pT]XX[E/D/**pS*/pY***] | Casein Kinase II substrate motif |  |
| 105 - 108 | SNSD | [pS/pT]XX[E/D] | Casein Kinase II substrate motif |  |
| 106 - 111 | NSDGAS | X[pS/pT]XXX[A/P/S/T] | G protein-coupled receptor kinase 1 substrate motif |  |
| 107 - 111 | SDGAS | pSXXX[pS/pT] | MAPKAPK2 kinase substrate motif |  |
| 107 - 111 | SDGAS | pSXXX**pS*** | GSK3 kinase substrate motif |  |
| 108 - 111 | DGAS | [E/D]XX[pS/pT] | Casein Kinase I substrate motif |  |
| 109 - 112 | GASP | XXpSP | GSK-3, ERK1, ERK2, CDK5 substrate motif |  |
| 110 - 112 | ASP | X[pS/pT]P | GSK-3, ERK1, ERK2, CDK5 substrate motif |  |
| 111 - 112 | SP | pSP | ERK1, ERK2 Kinase substrate motif |  |
| 111 - 113 | SPE | pSX[E/**pS*/pT***] | Casein Kinase II substrate motif |  |
| 113 - 116 | EPCT | [E/D]XX[pS/pT] | Casein Kinase I substrate motif |  |
| 117 - 119 | VTP | X[pS/pT]P | GSK-3, ERK1, ERK2, CDK5 substrate motif |  |

Results were obtained from PhosphoMotif Finder of the Human Protein Reference Database (<http://www.hprd.org/PhosphoMotif_finder>). Our data show that amino acids 1-216 of OCT4 are involved in *MYC* transcriptional activation. Thus, kinases that are associated with the motifs in amino acid 1-216 of OCT4 were studied. Phosphorylated residues are marked in red. * (blue) indicates the residues that must already be phosphorylated for the enzyme to recognize the motif.
