# Supplementary material for: MYC transcription activation mediated by OCT4 as a mechanism of resistance to 13-cisRA-mediated differentiation in neuroblastoma
Source: Cell Death Dis. 2020 May 14;11(5):368. doi: 10.1038/s41419-020-2563-4 (PMC7224192; doi:10.1038/s41419-020-2563-4)
Supplement: Supplementary file 4 — Suppl Table 4 [file 41419_2020_2563_MOESM4_ESM.docx]

**Supplementary Table 4**. List of Reagent and Resources used for the current study.

| REAGENT or RESOURCE | | | | | | SOURCE | | IDENTIFIER | |  |
| --- | --- | --- | --- | --- | --- | --- | --- | --- | --- | --- |
| Experimental models | | | | | |  | |  | |  |
| SMS-LHN (LHN) | | | | | | Cogcell.org | | N/A | |  |
| SMS-LHN-R (LHN-R) | | | | | | This paper | | N/A | |  |
| CHLA-15 | | | | | | Cogcell.org | | N/A | |  |
| CHLA-20 | | | | | | Cogcell.org | | N/A | |  |
| CHLA-78 | | | | | | Cogcell.org | | N/A | |  |
| CHLA-95 | | | | | | Cogcell.org | | N/A | |  |
| See Table 1 for the models obtained from Children’s Oncology Group Repository. | | | | | | | | | |  |
| Antibodies | | | | | |  | |  | |  |
| Anti-FLAG M2 | Sigma-Aldrich | | Cat# F3165, RRID: AB_259529 | | | | | | | |
| Anti-FLAG synthetic antibody for immunoprecipitation | Sigma-Aldrich | | Cat# F2426, RRID: AB_2616449 | | | | | | | |
| Anti-Nanog | Becton Dickinson | | Cat# 560109, RRID: AB_1645597 | | | | | | | |
| Anti-Cyclin A | Becton Dickinson | | Cat# 611269, RRID: AB_398797 | | | | | | | |
| Anti-CDK4 | Becton Dickinson | | Cat# C18720 | | | | | | | |
| Anti-TCF3 | Becton Dickinson | | Cat# 554199, RRID: AB_395298 | | | | | | | |
| Anti-OCT4 | Abcam | | Cat# AB19857, RRID: AB_445175 | | | | | | | |
| Anti-FGF4 | Thermo Fisher | | Cat# PA5-15464, RRID: AB_2102653 | | | | | | | |
| Anti-UTF1 | Thermo Fisher | | Cat# MA5-17197, RRID: AB_2538668 | | | | | | | |
| Anti-His | Thermo Fisher | | Cat# 70796, RRID: AB_11213479 | | | | | | | |
| Anti-myc | Thermo Fisher | | Cat# 46-1155 | | | | | | | |
| Anti-V5 | Thermo Fisher | | Cat# 46-1157 | | | | | | | |
| Anti-MYCN | Santa Cruz | | Cat# sc-791, RRID: AB_2251128 | | | | | | | |
| Anti-GAPDH | Santa Cruz | | Cat# sc-47724, RRID: AB_627678 | | | | | | | |
| Anti-LDH | Santa Cruz | | Cat# sc-27232, RRID: AB_10611212 | | | | | | | |
| Anti-Lamin A/C | Santa Cruz | | Cat# sc-20681, RRID: AB_648154 | | | | | | | |
| Anti-RARβ | Santa Cruz | | Cat#sc552 | | | | | | | |
| Anti-HA | Roche | | Cat# 11867423001, RRID: AB_10094468 | | | | | | | |
| Anti-DNA-PKcs | MBL International | | Cat# JM-3130-100, RRID: AB_591254 | | | | | | | |
| Anti-GST | MBL International | | Cat# M071-3, RRID: AB_591784 | | | | | | | |
| Anti-c-MYC | EMB Millipore | | Cat# MABE282, RRID: AB_11213164 | | | | | | | |
| Anti-ERα | EMB Millipore | | Cat# 06-935, RRID: AB_310305 | | | | | | | |
| Anti-NeuN | EMB Millipore | | Cat# MAB377, RRID: AB_2298772 | | | | | | | |
| Anti-SOX2 | EMB Millipore | | Cat# MAB4423, RRID: AB_11213224 | | | | | | | |
| Anti-MAPKAPK2 for immunoblotting | Cell Signaling | | Cat# 3042S, RRID: AB_10694238 | | | | | | | |
| Anti- Anti-pMK2^T222^ | Cell Signaling | | Cat#3316S | | | | | | | |
| Anti-p38α MAPK | Cell Signaling | | Cat#2917S | | | | | | | |
| Anti-pp38α MAPK^T180Y182^ | Cell Signaling | | Cat#9211S | | | | | | | |
| Anti-HSP27 | Cell Signaling | | Cat#2402S | | | | | | | |
| Anti-pHSP27^S78^ | Cell Signaling | | Cat#2405S | | | | | | | |
| Anti-MAPKAPK2 for IHC | Abnova | | Cat#H00009261, RRID: AB_ | | | | | | | |
| Anti-phospho-MK2^T334^ | Cell Signaling | | Cat# 3007S, RRID: AB_1532097 | | | | | | | |
| Anti-phospho-OCT4^S111^ | This paper | | RRID: AB_2721810 | | | | | | | |
| Oligonucleotides | | | | | |  | |  | |  |
| Biotin-c-MYC^-1209/-1140^ dsDNA WT probe:  Biotin- CTGTGCATACATAATGCATAATACA-  TGACTCCCCCCAACAAATGCAATGGGAGTTTATTCATAACGCGCT | | | | | | This paper | | N/A | |  |
| Biotin-c-MYC^-1209/-1166^ dsDNA WT probe:  Biotin- CTGTGCATACATAATGCATAATACATGACTCCCCCCAACAAATGC | | | | | | This paper | | N/A | |  |
| Biotin-c-MYC^-1173/-1145^ dsDNA WT probe:  Biotin- ACAAATGCAATGGGAGTTTATTCATAAC | | | | | | This paper | | N/A | |  |
| Biotin-c-MYC^-1209/-1140^ dsDNA Mut2 probe:  Biotin-CTGTGGATAGATAGCAGACAATAC-  GCAACTCCCCCCAACAAGACCAAATGGAGTTTATCGTCAACGCGCT | | | | | | This paper | | N/A | |  |
| ChIP -1367/-946  F: 5’-CAGCTCTGGAACAGGCAGACA -3’  R: 5’-GCTAAGGCTGGGGAAAGG-3’ | | | | | | This paper | | N/A | |  |
| ChIP -1367/-1026  F: 5’-CAGCTCTGGAACAGGCAGACA-3’  R: 5’-TTCAGAGCGTGGGATGTTAG-3’ | | | | | | This paper | | N/A | |  |
| ChIP -1223/-946  F: 5’-TCCAATCCAGATAGCTGTGC-3’  R: 5’-GCTAAGGCTGGGGAAAGG-3’ | | | | | | This paper | | N/A | |  |
| ChIP -1223/-1140  F: 5’-TCCAATCCAGATAGCTGTGC-3’  R: 5’-AGCGCGTTATGAATAAACTCCCA-3’ | | | | | | This paper | | N/A | |  |
| POU5F1-shRNA-2:  F:5'-CCGGTGGGAGTCCCAGGACATGAAATTCAAGAGATTTCATGTCCTGGGACCC  TTTTTTACGCGTG-3',  R: 5’-AATTCACGCGTAAAAAAGGAGTCCCAGGACATGAAATCTCTTGAATTTCATGT  CCTGGGACTCCCA-3’. | | | | | | This paper | | N/A | |  |
| Primers (for *MYCN* ^promoter^ and *MYC*^enhancer^ into the *DDK-c-MYC-mER^TM^* gene reporter) system) | | | | | |  | |  | |  |
| WT MYC^-1209/-1140^ (*Xba1-Xho1*/*BamH1*):  F: 5’-CGCCGCCTCTAGACTCGAGCTGTGCATACATAATGCATAA-3’  R: 5’-AGCCCGAAGGATCCAGCGCGTTATGAATAAACTCCCA-3’ | | | | | | This paper | | N/A | |  |
| MYCN^promoter^ (*Xho1*/*BamH1*):  F: 5’-GGCTTATACTCGAGATCTGTCTGTGTTTGAGCTGTCG-3’  R: 5’-GGAATGTAGGATCCAGGTCTGGGTTCTTGCAGAT-3’. | | | | | | This paper | | N/A | |  |
| Recombinant DNA | | | | | |  | |  | |  |
| Plasmid: *pBABE-MYC^439^-mER^TM^* | | | | | Dr. Trevor Littlewood | | N/A | | |  |
| Plasmid: *pCMV6-DDK-mER^TM^* | | | | | This paper | | N/A | | |  |
| Plasmid: *pCMV6-entry-mycDDK vector* | | | | | OriGene | | Cat#PS100007 | | |  |
| Plasmid: *pCMV/MYCN-mycDDK* | | | | | This paper | | N/A | | |  |
| Plasmid: *pMYCN^-1/-1098^/MYCN-mycDDK* | | | | | This paper | | N/A | | |  |
| Plasmid: *pCMV6-DDK-MYC^454^-mER^TM^* | | | | | This paper | | N/A | | |  |
| Plasmid: *pCMV6-DDK-MYC^439^-mER^TM^* | | | | | This paper | | N/A | | |  |
| Plasmid: *pCMV6-MYC^454Δ121-158^-mER^TM^* | | | | | This paper | | N/A | | |  |
| Plasmid: *pCMV6-DDK-MYC^454Δ72-209^-mER^TM^* (TAD plus MBIII deletions) | | | | | This paper | | N/A | | |  |
| Plasmid: *pCMV6-DDK-MYC^454V409D^-mER^TM^* (interaction site with MIZ-1) | | | | | This paper | | N/A | | |  |
| Plasmid: *pCMV6-TCF3-mycDDK* | | | | | OriGene | | Cat#RC215432 | | |  |
| Plasmid: *pCMV6-POU5F1-mycDDK* | | | | | OriGene | | Cat#RC211998 | | |  |
| Plasmid: *pCMV6-POU5F1^nt1-411^-mycDDK* (OCT4^aa1-137^) | | | | | This paper | | N/A | | |  |
| Plasmid: *pCMV6-POU5F1^nt1-441^-mycDDK* (OCT4^aa1-147^) | | | | | This paper | | N/A | | |  |
| Plasmid: *pCMV6-POU5F1^nt1-648^-mycDDK* (OCT4^aa1-216^) | | | | | This paper | | N/A | | |  |
| Plasmid: *pCMV6-POU5F1^nt1-879^-mycDDK* (OCT4^aa1-293^) | | | | | This paper | | N/A | | |  |
| Plasmid: *pCMV6-POU5F1^nt412-1080^-mycDDK* (OCT4^aa138-360^) | | | | | This paper | | N/A | | |  |
| Plasmid: *pCMV6-POU5F1^nt691-1080^-mycDDK* (OCT4^aa231-360^) | | | | | This paper | | N/A | | |  |
| Plasmid: *pCMV6-POU5F1^Δnt412-636^-mycDDK* (OCT4*^Δ^*^aa138-212^) | | | | | This paper | | N/A | | |  |
| Plasmid: *pCMV6-POU5F1^Δnt691-867^-mycDDK* (OCT4*^Δ^*^aa231-289^) | | | | | This paper | | N/A | | |  |
| Plasmid: *pCMV6-POU5F1^Δnt412-636/691-867^-mycDDK* (OCT4*^Δ^*^aa138-212/231-289^) | | | | | This paper | | N/A | | |  |
| Plasmid: *pCMV6-POU5F1^Δnt430-615/691-867^-mycDDK* (OCT4*^Δ^*^aa144-205/231-289^) | | | | | This paper | | N/A | | |  |
| Plasmid: *c-MYC* CRISPR/Cas9 KO plasmid (h) | | | | | Santa Cruz | | Cat# sc-400001 | | |  |
| Plasmid: *c-MYC* HDR plasmid | | | | | Santa Cruz | | Cat# sc-400001 | | |  |
| Plasmid: *pLenti-C-mycDDK-IRES-Puro* | | | | | OriGene | | PS100064 | | |  |
| Plasmid: *pLenti-DDK-MYC^439^-mER^TM^* | | | | | This paper | | N/A | | |  |
| Plasmid: *pLenti-DDK-MYC^454^-mER^TM^* | | | | | This paper | | N/A | | |  |
| Plasmid: *pLenti-DDK-MYC^454Δ121-158^-mER^TM^* | | | | | This paper | | N/A | | |  |
| Plasmid: *pLenti-DDK-MYC^454Δ72-209^-mER^TM^* | | | | | This paper | | N/A | | |  |
| Plasmid: *pLenti-DDK-MYC^454V409D^-mER^TM^* | | | | | This paper | | N/A | | |  |
| Plasmid: *pcDNA3.1D/V5-His-TOPO^C^* | | | | | Thermo Fisher | | Cat# K480001 | | |  |
| Plasmid: *pcDNA3.1-mLef1-V5-His6* | | | | | This paper | | N/A | | |  |
| Plasmid: *pLKO.1-puro TRC1.5 lentiviral vector* | | | | | Addgene | | Cat# 10878 | | |  |
| Plasmid: *pLKO.1-puro-eGFP (NT-shRNA)* | | | | | Sigma | | Cat# SJC005 | | |  |
| Plasmid: *pLKO.1-puro-POU5F1 shRNA 1-5* | | | | | This paper | | N/A | | |  |
| Plasmid: *pCMV6-AN-HA* | | | | | OriGene | | Cat# PS100013 | | |  |
| Plasmid: *pCMV6-HA-β-Catenin* | | | | | This paper | | N/A | | |  |
| Plasmid: *pGEX-4T1* | | | | | GE Health | | Cat# 28-9545-49 | | |  |
| Plasmid: *pGEX-4T-1-GST-POU5F1WT* (GST-OCT4) | | | | | This paper | | N/A | | |  |
| Plasmid: *pGEX-4T-1-GST-POU5F1S^93A^* (GST-OCT4S^93A^) | | | | | This paper | | N/A | | |  |
| Plasmid: *pGEX-4T-1-GST-POU5F1S^111A^* (GST-OCT4S^111A^) | | | | | This paper | | N/A | | |  |
| Plasmid: *pMetLuc2 Reporter vector* | | | | | Clonetech | | Cat# 631729 | | |  |
| Plasmid: *pMetLuc2-Control Vector* | | | | | Clonetech | | Cat# 631735 | | |  |
| Plasmid: *pMYC^-1209/-1140^/MetLuc* | | | | | This paper | | N/A | | |  |
| Plasmid: *pMYC^-1/-1889^/MetLuc* | | | | | This paper | | N/A | | |  |
| Plasmid: *pMYC^-1/-546^/MetLuc* | | | | | This paper | | N/A | | |  |
| Plasmid: *pMYC^-547/-1554^/MetLuc* | | | | | This paper | | N/A | | |  |
| Plasmid: *pMYC^-946/-1554^/MetLuc* | | | | | This paper | | N/A | | |  |
| Plasmid: *pMYC^-547/-945^/MetLuc* | | | | | This paper | | N/A | | |  |
| Plasmid: *pMYC^-946/-1223^/MetLuc* | | | | | This paper | | N/A | | |  |
| Plasmid: *pMYC^-1224/-1554^/MetLuc* | | | | | This paper | | N/A | | |  |
| Plasmid: *pMYC^-1209/-1140^/DDK-MYC-mER^TM^* WT2 | | | | | This paper | | N/A | | |  |
| Plasmid: *pSEAP2-Control Vector* | | | | | Clonetech | | Cat# 631717 | | |  |
| Plasmid: *pCW57-MCS1-2A-MCS2* | | | | | Addgene | | Cat# 71782 | | |  |
| Plasmid: *pCW57.1-MCS (EcoR1-Sgf1-Kpn1-Pme1-Mlu1 linker)* (Dox-inducible) | | | | | This paper | | N/A | | |  |
| Plasmid: *pCW57.1-POU5F1WT-mycDDK* (Dox-inducible) | | | | | This paper | | N/A | | |  |
| Plasmid: *pCW57.1-POU5F1S^111A^-mycDDK* (OCT4S^111A^) (Dox-inducible) | | | | | This paper | | N/A | | |  |
| Critical Commercial Assays | | | | |  | |  | | |  |
| QuikChange Site-Directed Mutagenesis Kit | | | | | Agilent | | Cat# 210518 | | |  |
| Panomics Nuclear Extraction Kit | | | | | Panomics | | Cat# AY2002 | | |  |
| Panomics EMSA Gel Shift kit | | | | | Panomics | | Cat# AY1000 | | |  |
| Panomics DNA/Protein Array refill kit | | | | | Affymetrix | | Cat# 1315 | | |  |
| Lenti-vpak Packaging Kit | | | | | OriGene | | Cat# TR30037P5 | | |  |
| Panomics Protein/DNA Combo Array | | | | | Affymetrix | | Cat# 1215 | | |  |
| Chromatin Shearing Optimization iDeal ChIP-Seq kit for transcription factors | | | | | Diagenode | | Cat# C01010055 | | |  |
| EZview Red Streptavidin Affinity Gels | | | | | Sigma-Aldrich | | Cat# F2426 | | |  |
| EZview Red anti-HA Affinity Gels | | | | | Sigma-Aldrich | | Cat# E6779 | | |  |
| Software and Algorithms | | | | | |  | |  | |  |
| GraphPad Prism v6 | | | | GraphPad Software | | | | | N/A | |
| FlowJo Software v7.6 | | | | Tree Star Inc. | | | | | N/A | |
| PhosphoMotif Finder | | http://whttp://www.hprd.org/PhosphoMotif_finder | | | | | | | |  |
