# Supplementary material for: MYC transcription activation mediated by OCT4 as a mechanism of resistance to 13-cisRA-mediated differentiation in neuroblastoma
Source: Cell Death Dis. 2020 May 14;11(5):368. doi: 10.1038/s41419-020-2563-4 (PMC7224192; doi:10.1038/s41419-020-2563-4)
Supplement: Supplementary file 5 — Suppl Figure Legends [file 41419_2020_2563_MOESM5_ESM.docx]

**Supplementary Figure legends**

**Supplementary Fig. 1. c-MYC for resistance mechanism to 13-*cis*RA.**

1. Gradual increase in the expression of c-MYC protein and decrease in expression of MYCN protein in LHN cells during selection for 13-*cis*RA resistance (Con: vehicle control (0.1% ethanol), C1=1^st^ 13-*cis*RA treatment, C3 the 3^rd,^ and C5 the 5^th^, Off2: 14 days off treatment after C1). The experiment was repeated to confirm the changes of proteins.
2. *DDK-MYC-ER^TM^* constructs encoding human wild-type MYC [AUG- (MYC439) or CUG-(MYC454)] fused with the hormone-binding domain of a modified murine estrogen receptor (mER^TM^), point substitution (*MYC454^V409D^*) and a deletion mutant (*MYC454^Δ72-209^*) were packaged into a pLenti-c-mycDDK-IRES-Puro vector and transduced into LHN cells. DDK = FLAG®, a trademark of Sigma Aldrich.
3. Nuclear expression of DDK-MYC-mER^TM^ and the MYC mutant constructs induced by 4-OHT (7.8 nM in ethanol for 24 h) in LHN cells. The expression of c-MYC (detected via anti-FLAG antibody) was confirmed by immunoblotting of cytosolic (C) and nuclear (N) fractions. The experiment was repeated to confirm the changes of proteins.
4. Nuclear localization and downstream Cyclin A activation effects of wild-type and mutant MYC when treated with 13-*cis*RA treatment. In LHN cells stably expressing MYC variants confirmed using the anti-FLAG antibody, Cyclin A expression was assessed after cells were treated with 13-*cis*RA for 14 days using immunoblotting of cytosolic (C) and nuclear (N) fractions of the cells. The experiment was repeated to confirm the changes of proteins.
5. Cell cycle analyses of LHN cells stably expressing wild-type MYC (MYC439 and MYC454) or its mutants (MYC454^V409D^ and MYC454^Δ72-209^) that were treated with vehicle only (blue, n=3) or 5 µM of 13-*cis*RA (red, n=3) for 14 days. Top: representative histograms, Bottom: Percentage of cells in each phase of cell cycle. The experiment was repeated twice.
6. Overexpression of the *MYC* in the LHN cells can suppress *MYCN* expression. The expression levels of MYCN and c-MYC proteins were examined in LHN cells stably expressing the exogenous DDK-mER^TM^ (Empty vector), MYC WT and its NTD deletion variant by infection with a lentivirus harboring *DDK-mER^TM^*, *DDK-MYC^454^-mER^TM^* or *DDK-MYC^454Δ121-158^-mER^TM^*. The LHN/MYC WT and LHN/MYC MT (ΔTAD) cells grown in 10-cm tissue culture plates were harvested after treatment with vehicle or 31.3 nM of 4-OHT for 24 h. Next, cells were performed with the subcellular fractionation and then the fractions (10 μg) extracted from the cytosolic (C) and nuclear extract (N) were run on 4-12% SDS-PAGE and followed by IB with the indicated antibodies. The experiment was repeated to confirm the results.

**Supplementary Fig. 2. Increased OCT4 protein in 13-*cis*RA resistant LHN-R neuroblastoma cells.**

1. Protein-DNA array-based identification of transcription factors (TFs) that are increased in LHN-R cells relative to LHN. Nuclear extracts prepared from 13-*cis*RA sensitive cells (LHN) and resistant cells (LHN-R) were obtained using Panomics’ Nuclear Extraction Kit. To systematically examine the potential cross-talk between MYC and multiple TFs simultaneously, the biotin-labeled DNA binding oligonucleotides were mixed and incubated with the nuclear extracts (15 mg). Next day, the probes in the protein/DNA complexes are extracted and hybridized to the Affymetrix combo protein/DNA array (spotted with 345 specific TF DNA-consensus sequences) for detection of the HRP-based ECL signals. The screening experiment was conducted twice.
2. TFs identified to be elevated or decreased in LHN-R cells relative to LHN. The TF’s with over 2-fold increase or 50% reduction were included. The quantitation used densitometry and the numbers were normalized by the controls, column 24 and row P.
3. Relative mRNA levels of *POU5F1* and *TCF3* in LHN-R (n=3) compared with LHN cells (n=3). The total mRNAs prepared from the LHN and LHN-R cells using RNAeasy Kits were subjected to real-time RT-PCR analysis of *POU5F1* and *TCF3* using the primers and probes listed in STAR ONLINE METHODS. The relative mRNA transcript levels were normalized using housekeeping gene *GAPDH*. ** p<0.01, ***p<0.001. The experiment was repeated twice.

**Supplementary Fig. 3. OCT4 binds to *MYC* promoter/enhancer region and validation of OCT4 as upstream of MYC transcription activation.**

1. Knockdown effects of *POU5F1* (left) and *TCF3* (right) by siRNA on c-MYC protein expression in LHN-R cells. The experiment was repeated twice.
2. Schematic representation of the *MYC^-1/-1899^/MetLuc* reporter gene and the constructs expressing different TFs. Transcriptional activity was assessed in a Ready-To-Glow secreted luciferase reporter assay system as described under Methods.
3. OCT4 binding to the proximal enhancer of the *MYC* gene. EMSA was used to analyze the interactions between the native OCT4 protein (0.5 mg) with a biotin-conjugated 44-bp (OBS1; *MYC^-1209/-1166^*), 29-bp (OBS2; *MYC^-1173/-1145^*), or 70-bp double-stranded DNA probe (OBS1+OBS2; *MYC^-1209/-1140^*) (20 fmol each) containing the binding elements of OCT4. The consensus sequences of the OCT4 binding elements used in this study were described in detail under STAR ONLIE METHODS. EMSA with the probe detected OCT4 complex including Mono-OCT4/Biotin-*MYC^-1173/-1145^*, Mono- and Di-OCT4/Biotin-*MYC^-1209/-1166^*, or Mono- and Di-OCT4/Biotin-*MYC^-1209/-1140^* dsDNA using HRP-conjugated streptavidin (1:3000) and followed by detection with the ECL system. The experiment was repeated twice.

**Supplementary Fig. 4. OCT4 as upstream of *MYC* transcription activation.**

1. The wild-type and the deletion mutants of OCT4 tagged with mycDDK epitopes at their COOH-termini were constructed.
2. DNA-binding domain(s) of OCT4 in the *MYC* promoter regions using *MYC^-1209/-1140^/DDK-MYC-mER^TM^* reporter gene in HEK293FT cells transfected with the indicated OCT4 constructs. ERa: c-MYC expression, DDK (FLAG): expression of exogenous DDK-tagged OCT4 variants. The experiment was repeated to confirm the results.

**Supplementary Fig. 5. C**hanges in MYCN by exogenous MYC expression and effect of MAPKAPK2 in 13-cisRA resistance and survival of neuroblastoma patients.

1. Effect of stable *MAPKAPK2* knockdown on OCT4 and c-MYC protein expression in COG-N-443h (established from a PD patient sample). The experiment was repeated to confirm the results.
2. Reversal of 13-*cis*RA resistance shown as neurite outgrowth in *MAPKAPK2* knock-down COG-N-443h cells. Cells stably transduced with non-targeting NT-shRNA or *MAPKAPK2*-shRNA were treated with vehicle control or 13-*cis*RA for 14 days. The changes in neurite outgrowth by MAPKAPK2-shRNA and 13-*cis*RA was confirmed twice. A scale bar: 200 μM.
3. Overall survival of patients by *MAPKAPK2* expression in neuroblastoma from the NCI TARGET database. Of the total patients (n=247), patients with *MYCN* non-amplification (n=175) were used for the analysis. Median expression of *MAPKAPK2* was used to compare the survival differences between high and low expression of *MAPKAPK2*.
4. Event-free survival of patients by *MAPKAPK2* expression in neuroblastoma from the NCI TARGET database. Of the total patients (n=247), patients with *MYCN* non-amplification (n=175) were used for the analysis. The data was scanned to identify maximum separation of the curves, and the *p* value was adjusted by Bonferroni adjustment.
5. Event-free survival of patients by *MAPKAPK2* expression in neuroblastoma from the NCI TARGET database. Of the total patients (n=247), patients with *MYCN* non-amplification (n=175) were used for the analysis. Median expression of *MAPKAPK2* was used to compare the survival differences between high and low expression of *MAPKAPK2*.

**Supplementary Fig. 6.** Expression and detection of human recombinant OCT4 wild-type, S93A and S111A mutants in bacterial system**.**

1. After IPTG induction, the GST and human recombinant GST-OCT4, GST-OCT4^S93A^ and GST-OCT4^S111A^ fusion proteins expressed in BL21/DE3 strain of *E. coli* before purification subjected to SDS-PAGE and stained with Coomassie brilliant blue solution. The experiment was repeated to confirm the results.
2. Proteins in **(a)** detected by immunoblotting using anti-OCT4 or anti-GST antibody. The experiment was repeated to confirm the results
3. Assessment of DNA binding ability of wild-type OCT4 and mutant OCT4^S111A^ using *MYC^-1209/-1140^/Luc* reporter assay system. Empty vector, *POU5F1-mycDDK* and *POU5F1^S111A^-mycDDK* (4 μg each) were separately co-transfected with reporter gene *MYC^-1209/-1140^/Luc* (4 μg) in HEK293FT cells (n=6, p <0.01). After 48 h, the protein lysates (20 μg) were run and analyzed by WB using specific antibodies, as indicated. Luciferase activity assay was measured using Ready-To-Glow^TM^ Secreted Luciferase Reporter System. The experiment was repeated to confirm the results.

**Supplementary Fig. 7.** pMK2T222 expression in Dx and PD cell lines of neuroblastoma and upstream of MK2 phosphorylation.

a&b The levels of pMK2^T222^ phosphorylation are higher in patient-derived neuroblastoma cell lines PD than in Dx. The protein lysates (20 μg) were run and analyzed by SDS/PAGE and IB using specific antibodies, as indicated. CHLA-20 was used as a positive control for protein normalization. The experiment was repeated to confirm the results.

1. Activation of the p38 MAPK kinase was critical for driving the MK2/OCT4/MYC pathway in two NB cell lines, COG-N-508h and COG-N-514. Hyper activation of p38 MAPK at residues of Thr180 and Tyr182 not only can increase the levels of pMK2^T222^ and its downstream target pHSP27^S78^ but also pOCT4^S111^, c-MYC and Cyclin A. Inhibition of the p38 activity by p38 specific inhibitor SB203508 (10 μM) can reduce their protein expression levels, suggesting that activation of the MK2/OCT4/MYC axis is p38 dependent. The experiment was repeated to confirm the results.
2. There was no changed in the expression levels of the retinoid receptor β when compared the 13-*cis*RA sensitive (LHN) with resistant (LHN-R) NB cell lines. The protein lysates (20 μg) were analyzed by SDS/PAGE and IB using specific antibodies. CHLA-20 and LHN-R were acted as positive controls for c-MYC protein overexpression. The experiment was repeated to confirm the results.
