# Supplementary figures and images for: MYC transcription activation mediated by OCT4 as a mechanism of resistance to 13-cisRA-mediated differentiation in neuroblastoma
Source: Cell Death Dis. 2020 May 14;11(5):368. doi: 10.1038/s41419-020-2563-4 (PMC7224192; doi:10.1038/s41419-020-2563-4)

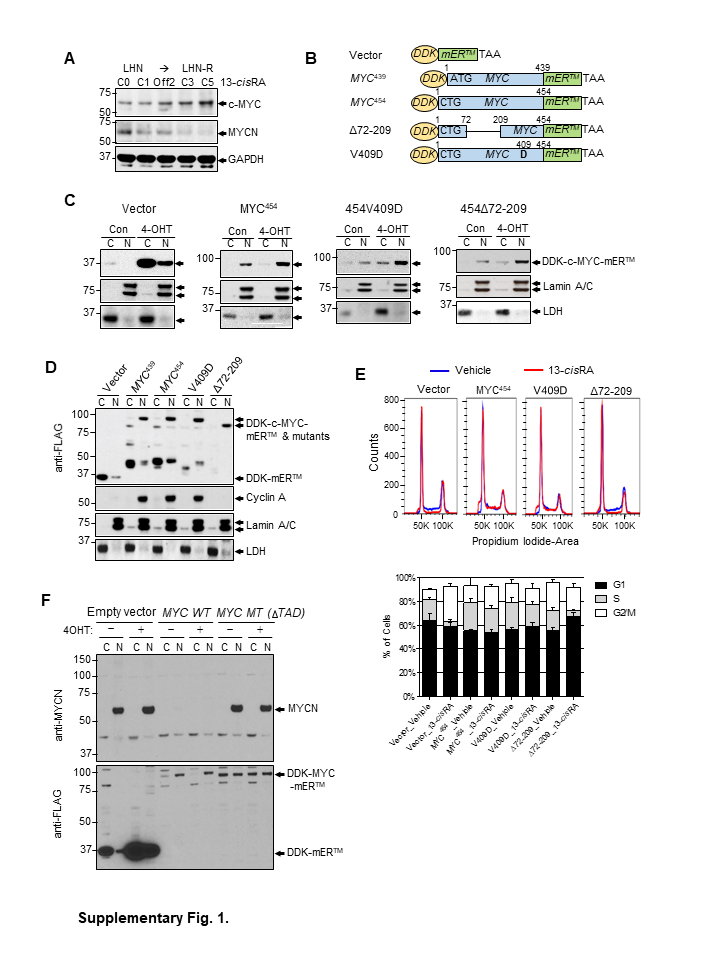

Supplement: Supplementary file 6 — Suppl Figure 1 [file 41419_2020_2563_MOESM6_ESM.tif]

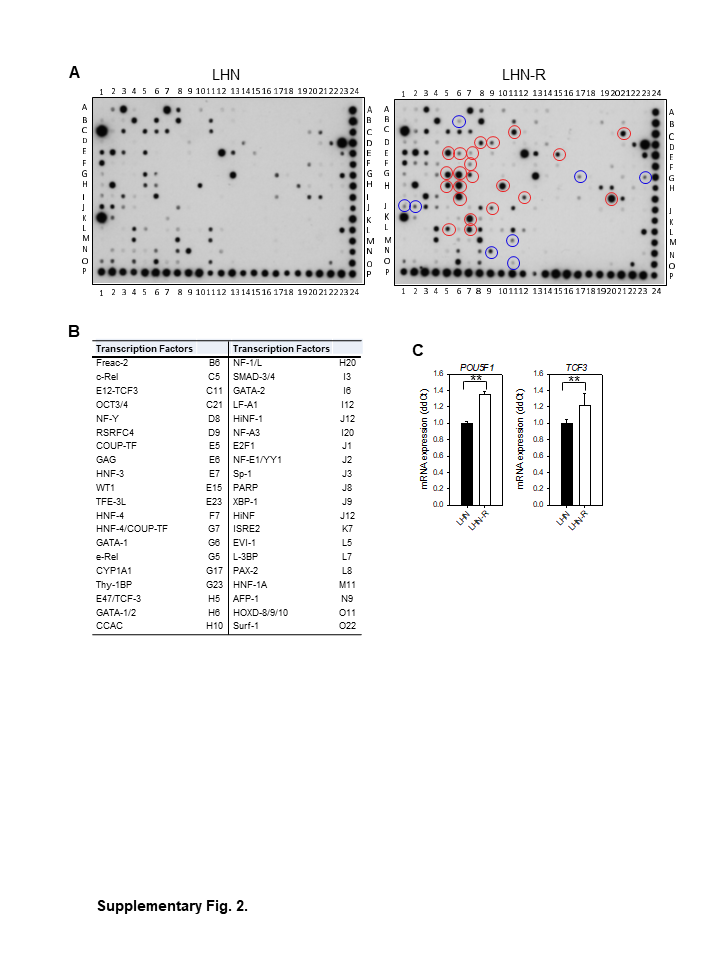

Supplement: Supplementary file 7 — Suppl Figure 2 [file 41419_2020_2563_MOESM7_ESM.tif]

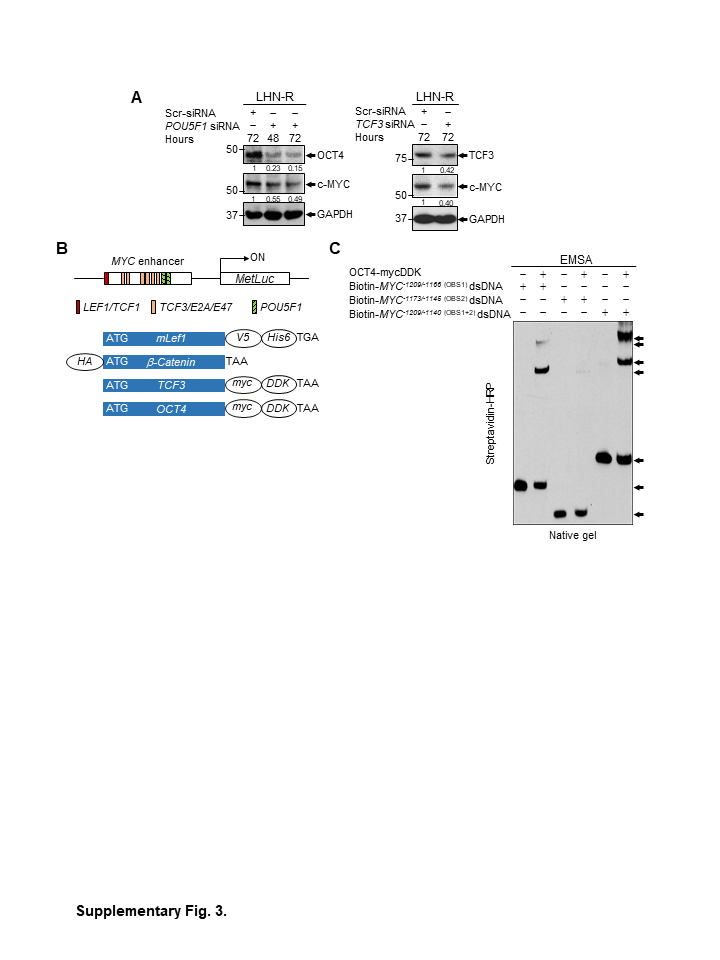

Supplement: Supplementary file 8 — Suppl Figure 3 [file 41419_2020_2563_MOESM8_ESM.tif]

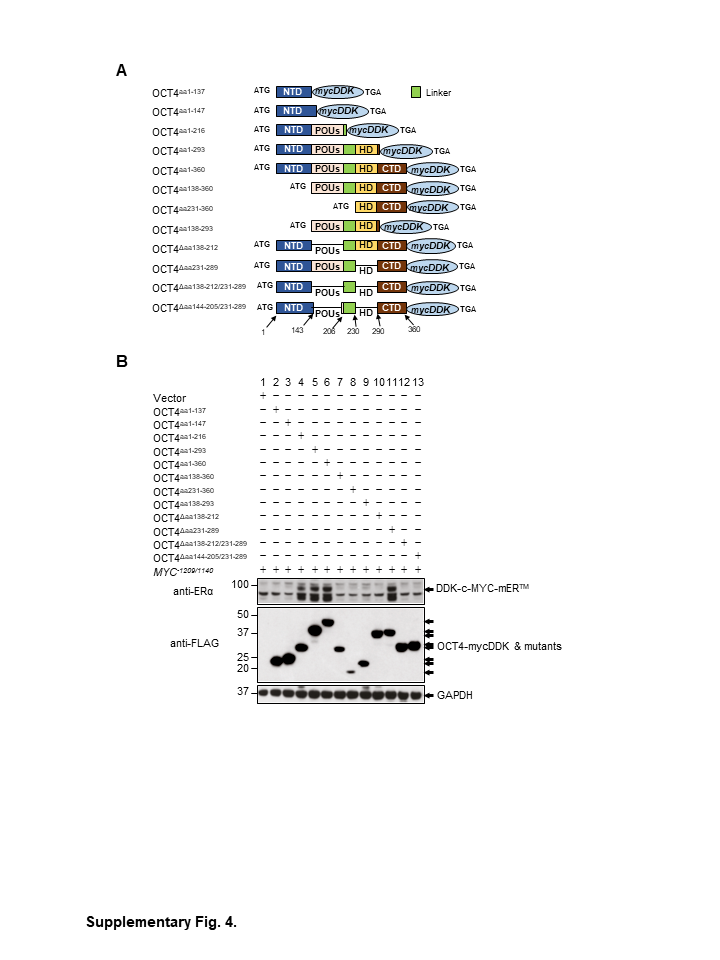

Supplement: Supplementary file 9 — Suppl Figure 4 [file 41419_2020_2563_MOESM9_ESM.tif]

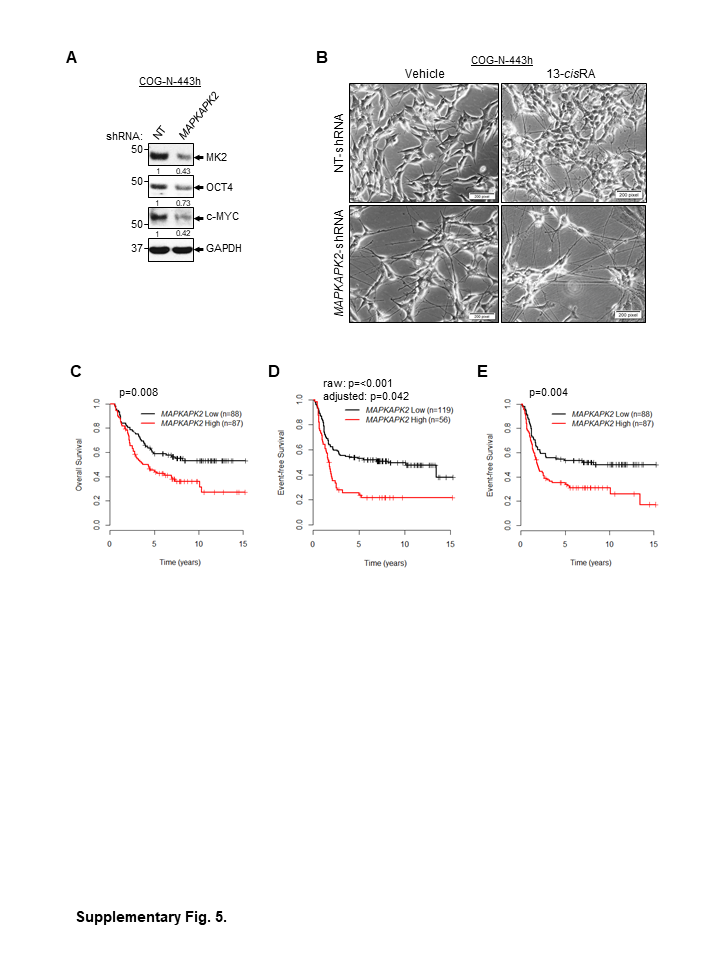

Supplement: Supplementary file 10 — Suppl Figure 5 [file 41419_2020_2563_MOESM10_ESM.tif]

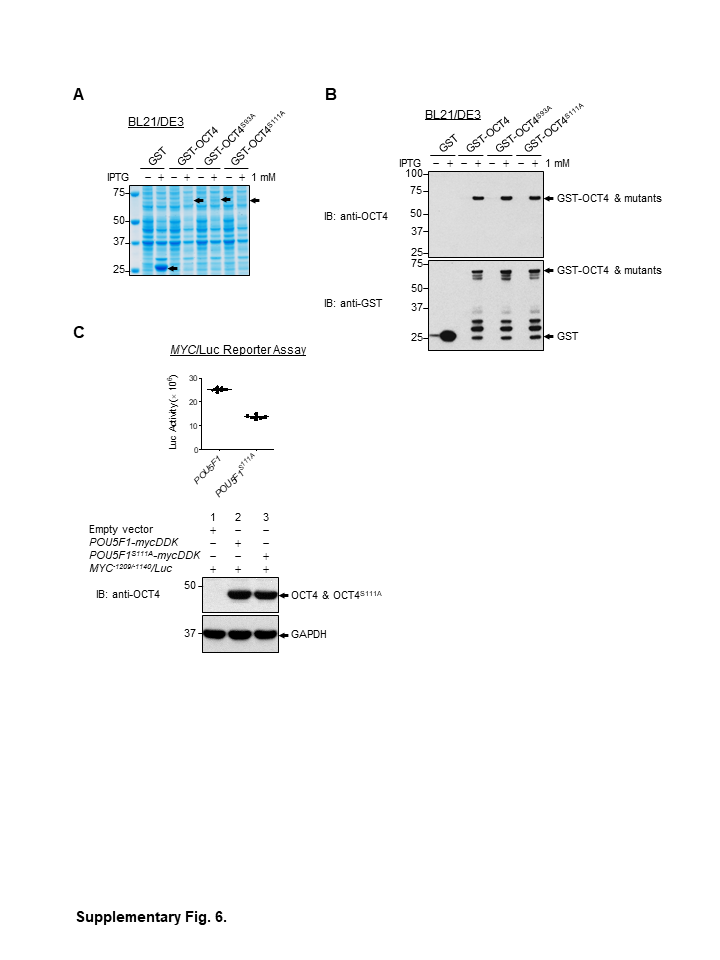

Supplement: Supplementary file 11 — Suppl Figure 6 [file 41419_2020_2563_MOESM11_ESM.tif]

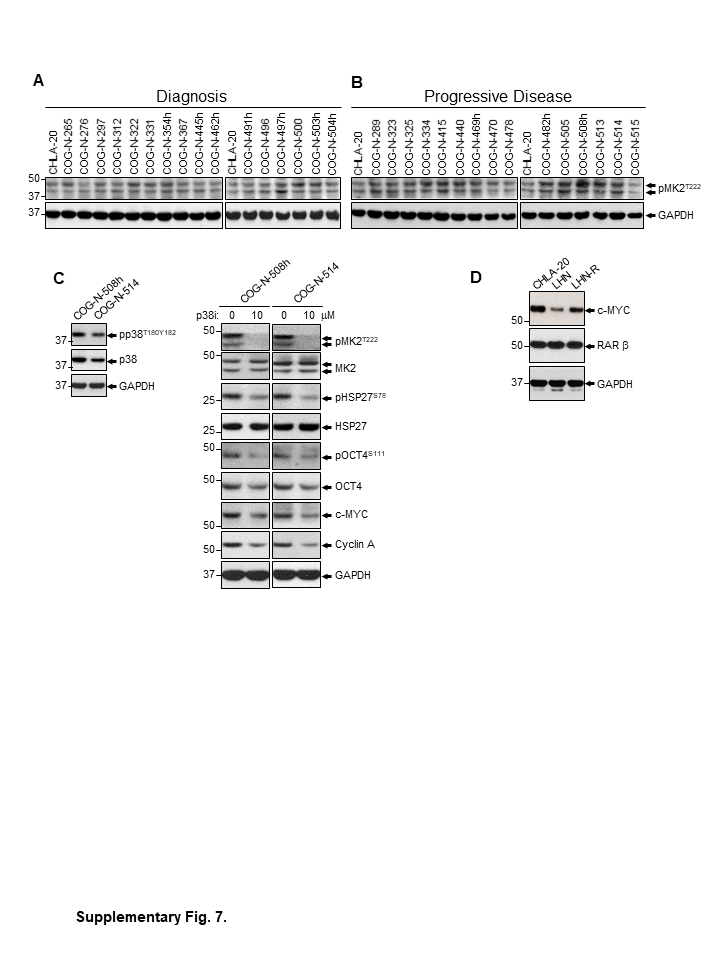

Supplement: Supplementary file 12 — Suppl Figure 7 [file 41419_2020_2563_MOESM12_ESM.tif]
